# High-Pressure Polymorphs Nucleated and Stabilized by Rational Doping under Ambient Conditions

**DOI:** 10.1021/acs.jpcc.1c07297

**Published:** 2021-10-19

**Authors:** Fatemeh Safari, Andrzej Katrusiak

**Affiliations:** Faculty of Chemistry, Department of Materials Chemistry, Adam Mickiewicz University, Uniwersytetu Poznańskiego 8, 61-614 Poznań, Poland

## Abstract

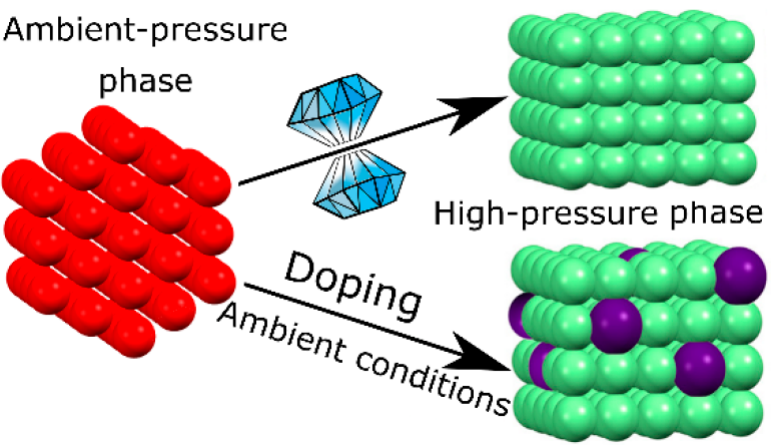

High-pressure polymorphs can be obtained and stabilized at ambient pressure by utilizing dopants with more voluminous molecules, inducing internal strain in the structures. This effect has been confirmed for doped resorcinol and imidazole derivatives by nucleating and stabilizing their high-pressure phases under ambient conditions. The dopant molecular volume and concentration, as well as the bulk modulus of the polymorph in the binary system, are related to the stability region in the single-component phase diagram. High-pressure isothermal and isochoric recrystallizations yielded pure single crystals of resorcinol ε above 0.20 GPa and a new polymorph ζ above 0.70 GPa. These recrystallizations of pure resorcinol revealed within 1 GPa of the *p*–*T* phase diagram the boundaries and the stability regions of four resorcinol polymorphs α, β, ε, and ζ, contrary to the compression experiments on ambient-pressure polymorphs α and β, when the high-pressure phases were hidden behind the wide hysteresis extending to nearly 5 GPa. The hysteresis, originating from the H-bonding networks, hinders the formation of polymorphs ε and ζ when polymorphs α and β are compressed without melting or dissolving the crystals. Polymorph ζ is the only known resorcinol structure built of hydrogen-bonded layers.

## Introduction

The wide variety of properties displayed by the same chemical compound in its different forms, such as polymorphs, glasses, size-scaled (nano)particles, and epitaxial layers, has stimulated research aimed at obtaining new materials desired for innovative and challenging applications. For example, studies on polymorphs of organic compounds have improved the performance of active pharmaceutical ingredients (APIs);^1−5^ polymorphs of photovoltaic materials, such as hybrid and purely inorganic perovskites, can cause undesired effects but also improve the performance of solar cells.^6^ Many methods for obtaining new polymorphs^7,8^ and for their theoretical prediction^9,10^ have been described. Among others, high pressure has been recognized as a very efficient tool for obtaining novel polymorphs and solvates of various compounds,^11−13^ such as paracetamol,^14^ urea,^15,16^ sucrose,^17^ benzimidazole,^18^ and others.^19,20^ However, most of the new forms obtained under high pressure and high/low temperature are unstable under normal conditions, which limits their practical applications. Here, we describe a simple method for obtaining and stabilizing high-pressure polymorphs under ambient conditions. These effects can be achieved by rationally doping a compound. In fact, the method of doping for obtaining new polymorphs is well-known;^21,22^ however, it was not connected to and optimized for specific regions of phase diagrams. The mechanism behind this phenomenon has been explained and verified for several compounds, but this study was inspired by intriguing inconsistencies in the phase diagram of resorcinol^23^ and by the recent discovery of polymorph ε obtained by mixing resorcinol with tartaric acid.^24^

Resorcinol is an important chemical agent^25,26^ and the first organic compound for which, in the 1930s, the structures of two polymorphs were determined.^27−29^ Back then, the density of low-temperature polymorph α being lower than that of high-temperature polymorph β and their space-group symmetry type *Pna*2_1_ (*Z* = 4) being the same despite considerable structural differences were counterintuitive. Since then, high-pressure polymorphs γ (space group *Pnna*) and δ (space group unknown)^30,31^ have also been postulated, but their structures have not been reported. Most recently, polymorph ε (space group *P*2_1_2_1_2_1_) was found in the mixture with polymorph β obtained by freezing the melt of resorcinol with the addition of tartaric acid in the form of a thin film.^24^ The structure of polymorph ε was determined from multicomponent powder X-ray diffraction (PXRD) combined with density functional theory (DFT) and molecular dynamics (MD) calculations. This discovery, quite puzzling after over a century of studies on this textbook example of polymorphs,^2^ raised a number of questions, such as the availability of pure resorcinol polymorph ε, its stability region in the *p*–*T* phase diagram, the role of the dopant, and more general questions about metastability, detection of the total energy-minimum forms, and the roles of symmetry-independent units (*Z*′) and molecular conformation. Undoubtedly, McCrone’s famous statement “... that every compound has different polymorphic forms, and that, in general, the number of forms known for that compound is proportional to the time and money spent in research on that compound”^32^ has also been validated for resorcinol. Recent extensive studies on resorcinol as a function of temperature and pressure by X-ray and neutron diffraction, solid-state NMR, free-induction decay (FID) analysis, Raman spectroscopy, and other methods identified polymorph β as the high-pressure form of resorcinol up to 5 GPa, where it transforms to phase γ.^30,31^ Polymorph α could be isothermally compressed to 4 GPa; however, in other experiments, it transformed to polymorph β at 0.5 GPa.^31^ Recently, we investigated the structural origin of the pressure- and temperature-induced transformation between resorcinol phases α and β;^23^ two pressure ranges favoring the formation of resorcinol hydrates were also identified.^33^

Presently, we have established that polymorph ε is the stable form of pure resorcinol from 0.20 to 0.70 GPa, at which point another polymorph ζ becomes stable. Single crystals of polymorphs ε and ζ were grown, and their structures were determined. The limiting pressure of 0.20 GPa, where single crystals of polymorph ε obtained from pure resorcinol immediately transformed to polymorph α, was most surprising in the context of the prolonged handling of polymorph ε obtained from the mixture with tartaric acid.^24^ We have proposed a microstructural mechanism of doping stabilization of polymorph ε, and we have shown that this simple method also stabilizes high-pressure polymorphs of other compounds.

## Experimental Section

### High-Pressure Crystallizations

High-pressure experiments were performed in a diamond anvil cell (DAC)^34^ modified by mounting the anvils directly on the steel supports with conical windows. Gaskets were made of steel foil 0.2 mm thick with a spark-eroded hole 0.35 mm in diameter. Polymorph ε nucleated above 0.20 GPa from the solution of resorcinol in a methanol/water 1:1 (vol) mixture (Figure S1). In another series of experiments, polymorph ε nucleated and grew in the form of a single crystal from the aqueous solution in the presence of resorcinol monohydrate (Figure S2): first, the monohydrate crystal was grown in the DAC at 0.80 GPa, and after releasing the pressure to 0.35 GPa, it started to dissolve while another crystal nucleated. After further reduction of the pressure to 0.20 GPa, the monohydrate dissolved completely, and a crystal of ε-resorcinol was isothermally grown when the pressure was slowly increased up to 0.25 GPa (Figure S2); the crystal grew when the pressure was increased to 0.50 GPa, and then no change in the size or shape was noticed. On average, the crystallization of one single-crystal sample from its nucleation to the final equilibration of growth at room temperature required about 1–3 h of the controlled microscopic experiment. The crystals were studied in situ by single-crystal X-ray diffraction (SCXRD).

Characteristically, polymorphs α, β, and ε could be isothermally compressed to over 1 GPa, while high-pressure recrystallization between 0.20 and 0.70 GPa resulted in polymorph ε only. Above 0.70 GPa, a new polymorph ζ of pure resorcinol was crystallized from the saturated solution in MeOH/EtOH/H_2_O (16:3:1) by the isochoric method (Figure S3). The SCXRD data were measured for this sample, and then again for the crystal isothermally compressed to 0.83, 1.00, and 1.20 GPa.

The pressure in the DAC chamber was calibrated by the ruby-fluorescence method before and after each diffraction measurement by using a Photon Control Inc. spectrometer of increased resolution, affording an accuracy of 0.02 GPa.^35,36^ A KUMA KM4-CCD diffractometer was used for SCXRD measurements. Data collection^37^ and preliminary reduction of data after correcting the intensities for the effects of DAC absorption, sample shadowing by the gasket, and sample absorption were performed.^38,39^ The structures were refined with full-matrix least-squares on *F*^2^ using SHELX-L.^40,41^ The crystallographic and experimental details are given in Table S1 and are deposited in CIF format in the Cambridge Structural Database with CCDC numbers 2084039–2084046.

### Sample Doping by Melting

Samples of 4.1 mg of ambient-pressure polymorphs (α-phase) of selected compounds listed in Table 2 were mixed by grinding with a dopant at 5, 15, and 25 wt % ratios and recrystallized by freezing the melt, following the procedure reported for α-resorcinol mixed with d-tartaric acid (d-Ta) by Zhu et al.^24^ Each of these mixtures, as well as the reference sample of the pure compound, was heated on the thermal stage of a microscope until all crystal grains were molten, after which the samples were left to cool down to room temperature and recrystallize (Figure S9). The cooling of the molten mixture from ca. 370–300 K took about 10 min, but the molten sample froze in few seconds. Subsequently, the samples were characterized by PXRD using a Bruker D8 diffractometer operating in the θ–2θ geometry with a Johansson monochromator, λ(Cu Kα_1_) = 1.54060 Å, and a LynxEye silicon-strip detector. The PXRD patterns were compared with the reference patterns calculated for the pure-compound phases as well as the applied dopant.^42,43^ In this way, the polymorphs constituting the samples were identified, and their ratio was established by comparing the intensities of reflections.

**Table 1:** Selected Crystallographic Data of Resorcinol Polymorphs α, β, ε, and ζ (cf. Tables S1 and S2) as Well as the Torsion Angles Describing the Molecular Conformation and H-Bond Directions

polymorph	α	β	ε	ε	ζ	ζ
pressure (GPa)	0.80(2)	0.91(2)	0.25(2)	0.96(2)	0.70(2)	1.2(2)
space group	*Pna*2_1_	*Pna*2_1_	*P*2_1_2_1_2_1_	*P*2_1_2_1_2_1_	*P*2_1_/*c*	*P*2_1_/*c*
unit cell *a* (Å)	10.2830(19)	7.5918(6)	17.876(5)	17.700(6)	10.6348(8)	10.465(3)
*b*	9.1431(7)	12.629(15)	10.464(6)	10.094(3)	9.5004(16)	9.425(5)
*c*	5.5953(3)	5.3029(14)	5.7045(16)	5.6096(6)	10.873(2)	10.787(6)
β (deg)	90	90	90	90	114.713(15)	113.70(4)
vol (Å^3^)	526.06(11)	508.4(6)	1067.0(8)	1002.2(5)	997.9 (3)	974.1(9)
*Z*/*Z*′	4/1	4/1	8/2	8/2	8/2	8/2
*D*_*x*_ (g/cm^3^)	1.390	1.439	1.371	1.459	1.446	1.502
conformer^a^	anti–anti	anti–syn	anti–syn	anti–syn	anti–syn	anti–syn
C2–C1–O1–H (deg)^b^	173	–164	–151/–127	–152/–128	–163/156	–163/155
C2–C3–O3–H (deg)	–170	–17	–3/45	10/48	–1.6/19	5/17
C2–C1–O1···O3 (deg)	170.3	–161.5	–148/–128	–136/–128.5	–149.5/146	–150/145.35
C2–C3–O3···O1 (deg)	–163.3	–25.4	6/58	0.8/62.23	6.8/29.8	7.17/31.03

a The hydroxyl group conformation is associated with torsion angles C2–C1–O1–H and C2–C3–O3–H3.

b The values for symmetry-independent molecules A and B, in polymorphs ε and ζ, are separated with the slash.

**Table 2:** List of Selected Host Samples and Compounds Used as Dopants

host compound	dopant
resorcinol	l-tartaric acid (l-Ta)
resorcinol	dl-tartaric acid (dl-Ta)
resorcinol	d-tartaric acid (d-Ta)
resorcinol	2-methyl benzimidazole (M-BzIm)
resorcinol	5,6-dimethylbenzimidazole (dM-BzIm).
imidazole (Im)	2-methyl benzimidazole (M-BzIm)
benzimidazole (BzIm)	2-methyl benzimidazole (M-BzIm)
benzimidazole (BzIm)	5,6-dimethylbenzimidazole (dM-BzIm)
2-methyl benzimidazole (M-BzIm)	5,6-dimethylbenzimidazole (dM-BzIm)

### Milling Mixed Samples

Preparation of doped samples, analogous to those obtained in the melting and freezing process, by milling mixtures of 4.1 mg of the ambient-pressure polymorph with the dopants listed in Table 2 at 5, 10, 15, 20, and 25 wt % was attempted. The samples were milled in hardened steel containers with several steel balls at a frequency of 30 Hz for 4 h. An MM 400 mill was used for all the powder samples. The subsequent PXRD patterns showed no changes in the ambient-pressure components in the mixtures, and no high-pressure polymorphs were detected.

## Results and Discussion

### High-Pressure Crystallization

We used the methods of in situ isothermal recrystallization and isochoric recrystallization to explore the stability regions of resorcinol polymorphs under pressure. The crystallization conditions were additionally varied by changing the solvent (methanol, ethanol, water, and their various mixtures), concentration of resorcinol, and temperature and pressure region chosen for nucleating and growing crystals. The crystallization was performed slowly to prevent kinetic nucleation and to obtain high-quality single crystals; their morphology and high-accuracy XRD data were used for identifying the polymorphs. The crystallization process and transformations of the sample crystals and their compression and decompression were observed through a microscope. In this way, we could repetitively obtain and identify any of the four polymorphs α, β, ε, and ζ of pure resorcinol in the pressure region up to 1.20 GPa (Table 1).

High-quality single crystals of polymorph ε were grown in isochoric and isothermal conditions between 0.20 and 0.70 GPa (Figures S1 and S2). Upon releasing the pressure, the ε crystals were crushed into small pieces below 0.20 GPa due to the strain induced by the first-order phase transition to polymorph α, identified by PXRD both for the sample kept below 0.20 GPa in the DAC and for the powder recovered to ambient conditions. The single crystals of polymorph ε could be compressed to 1 GPa, at which they were crushed. By performing in situ PXRD measurements, we confirmed that the tiny fragments are still in the ε phase. The damage was due to mechanical reasons caused by anisotropic strain in the elongated crystal samples bridging the opposite edges of the steel gaskets (Figures S1 and S2), which plastically deformed when the pressure in the DAC was increased. All recrystallization procedures above 0.70 GPa resulted in crystals of a new polymorph ζ (Figure S3). All these observations show that polymorph ε of resorcinol is stable between 0.20 and 0.70 GPa and that polymorph ζ is stable at still higher pressure.

**Figure 1:**
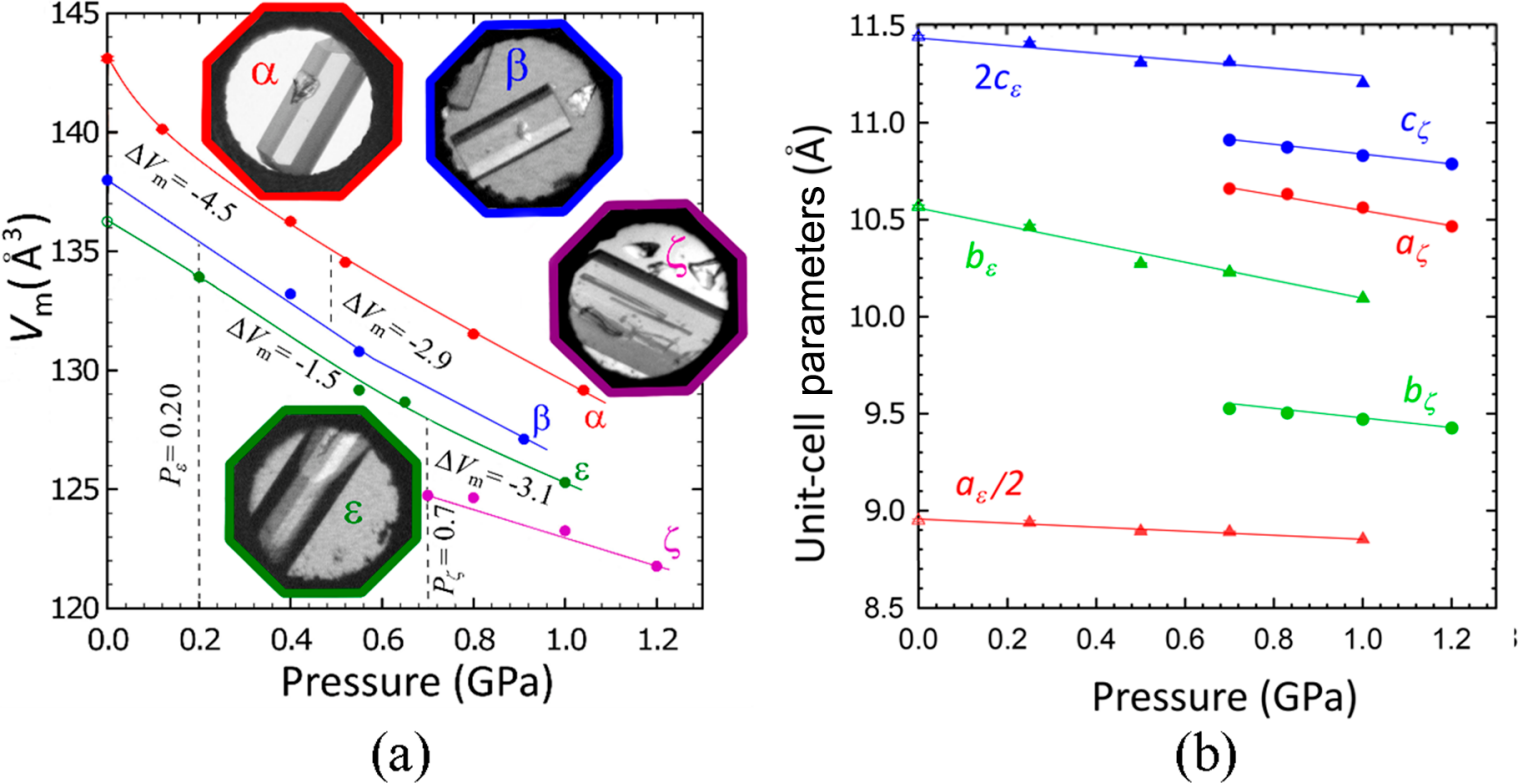
(a) Molecular volume (*V*_m_ = *V*/*Z*) of resorcinol polymorphs α, β, ε, and ζ plotted as a function of pressure. All ESDs are smaller than the plotted symbols. The empty symbol represents the ε phase obtained from a mixture of resorcinol with tartaric acid at 0.1 MPa (this data point was determined in ref (24)). The insets show single crystals of polymorphs grown in situ in the DAC (cf. Figures S1–S3). (b) Unit-cell parameter as a function of pressure for polymorph ε (triangles) and polymorph ζ (circles); parameters *a*_ε_ and *c*_ζ_ have been divided and multiplied by 2, in order to accommodate all plots in one drawing.

**Figure 2:**
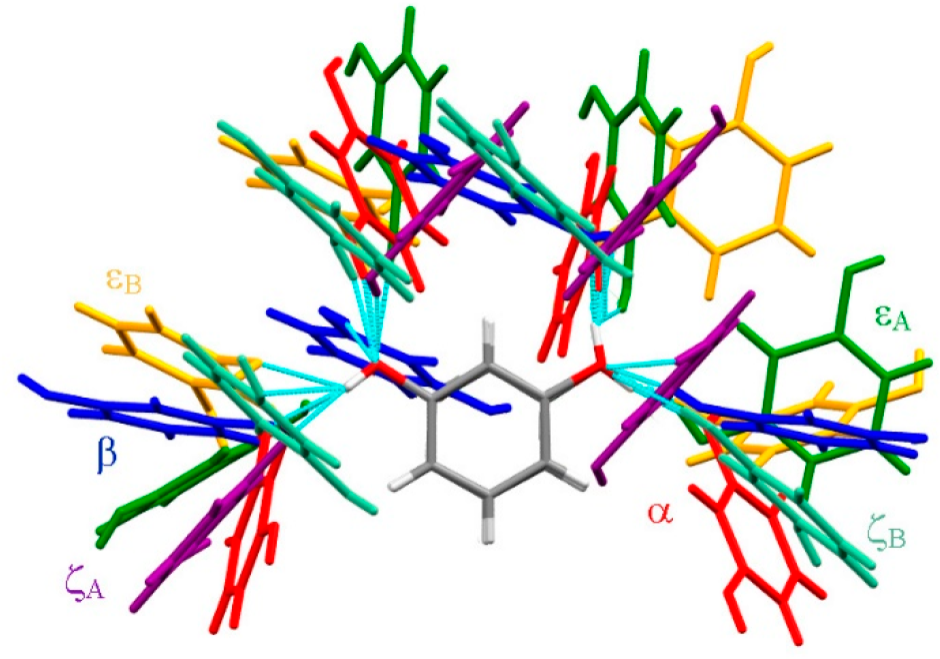
H-Bonded neighbors around the exactly superimposed central molecule (gray) in resorcinol polymorphs: α (red), β (navy blue), ε (green around molecule A and yellow around molecule B), and ζ (purple around molecule A and fern around molecule B). H-Bonds are indicated in cyan.

**Figure 3:**
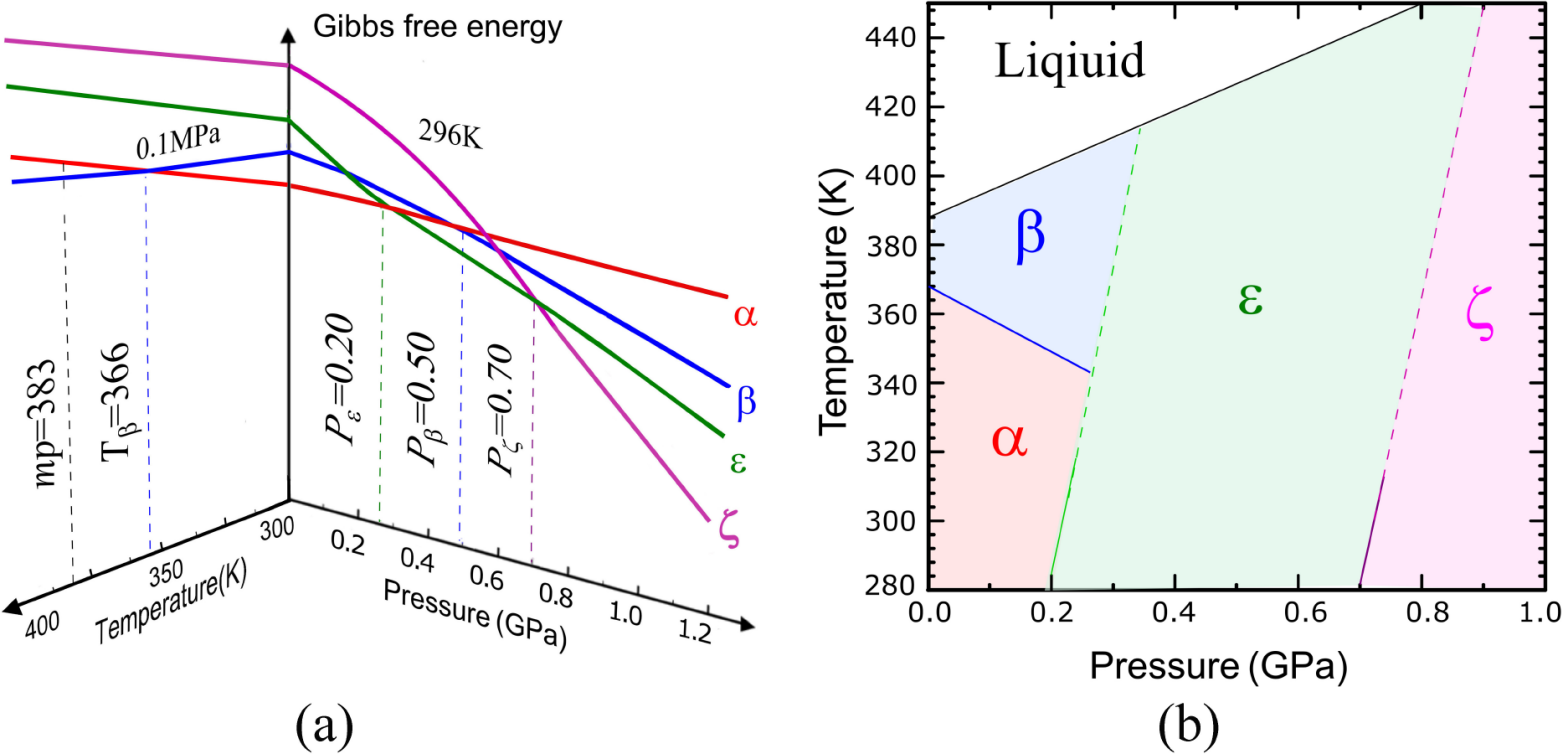
(a) Schematic diagram of the Gibbs free energy function vs pressure at 296 K and vs temperature at 0.1 MPa for resorcinol polymorphs α, β, ε, and ζ. Minimum pressure values for crystallizing polymorphs ε (*P*_ε_) and ζ (*P*_ζ_) are indicated; *P*_β_ marks the postulated equilibrium boundary pressure between polymorphs α and β in their metastable region. (b) *p*–*T* phase diagram of resorcinol; the dashed lines indicate phase boundaries extrapolated beyond those determined at 296 K in this study.

High-pressure SCXRD structural studies on polymorph ε showed that its molecular volume is smaller than those of polymorphs α and β in the all pressure ranges from 0.1 MPa to 1 GPa (Table 1, Figure 1a), but above 0.70 GPa, polymorph ζ is significantly denser than polymorph ε. This information corroborates the conclusions on the stability regions of polymorphs ε and ζ drawn from the high-pressure recrystallization, microscopic observations, and SCXRD/PXRD studies (Figure 1 and Figure S4). The *p*–*T* phase diagram outlined by us (Figure 3b) is consistent with the previous observations reported in the literature, with the reservation that, previously, the solid polymorphs α and β were only compressed and not dissolved. In those experiments, polymorphs α and β persisted to 5 GPa^23,30,31^ owing to the molecular conformations (Figure 2, Table 1) coupled to H-bonding networks (Table 1, Figure S6), preserving the present forms, although alternative structures could acquire a lower Gibbs free energy. This behavior, responsible for the extensive hysteresis,^44^ clearly corresponds to different Gibbs functions associated with the polymorphs, as illustrated in Figure 3a. These results compared to those obtained in Kahr’s group^24^ led us to the conclusion that high hydrostatic pressure causes some effects similar to those of doping resorcinol with tartaric acid, which results in the crystallization of polymorph ε at 0.1 MPa. Because of the instability of resorcinol ε at ambient pressure, we could not determine its unit-cell dimensions at 0.1 MPa, and in Figure 3, the dimensions previously determined by Kahr’s group^24^ for resorcinol doped with tartaric acid are plotted.

Apart from the lowest molecular volume (the highest density), the unique features of polymorph ζ—among the group of structurally determined resorcinol polymorphs α, β, ε, and ζ—are the centrosymmetric space group (Table 1) and that, in the crystal structure of ζ-resorcinol, the molecules are H-bonded into layers, which contrasts with the three-dimensional H-bonded networks in the other polymorphs (Figures S6–S8). It is remarkable that the compression of the layered structure of polymorph ζ is hardly anisotropic, as illustrated by the compression of the unit-cell parameters in Figure 1b. The polymorphs also differ in the conformations of hydroxyl groups, approximately described as either anti–anti (polymorph α) or anti–syn (polymorphs β, ε, and ζ). The descriptors “anti” and “syn” refer to the positions of ideally located hydroxyl hydrogen atoms with torsion angles C2–C1–O1–H and C2–C3–O3–H equal to 180° or 0°, respectively. The largest distortions from these ideal values are present in polymorph ε (Table 1). The hydroxyl group conformation indicates the direction of the H-bonds to the neighboring molecules (Figures S6–S8). The orientation of these close neighbors is further varied by torsion angles C–O···O–C (Figure 2). Undoubtedly, there are no easy paths for the molecules to change their arrangements and aggregation topologies by way of solid-to-solid phase transitions.

The polymorphic landscape of resorcinol as a function of pressure and temperature can be described by four Gibbs free energy functions shown in Figure 3a, and the *p*–*T* diagram in Figure 3b contains four polymorphs of resorcinol within the pressure range of 0–1 GPa. These results show that, at 300 K, polymorph β is metastable in all ranges of investigated pressure. Consequently, the recrystallization experiments yield the sequence of polymorphs α, ε, and ζ.

Owing to the wide hysteresis of resorcinol polymorphs, their volume compressibility β_v_ = −(1/*V*) d*V*/d*p*|_*T*=296 K_ could be independently determined at 0.1 MPa at 296 K (Figure 1a): for polymorph α the compressibility, β_v_, is 0.140 GPa^–1^; for polymorph β it is 0.091 GPa^–1^; for polymorph ε it is 0.081 GPa^–1^; for polymorph ζ the compressibility could be measured only above 0.7 GPa where it is equal to 0.048 GPa^–1^. This significantly smaller β_v_ for polymorph ζ results from (i) the clearly nonlinear compressibility decreasing with increasing pressure for molecular crystals, as well as (ii) the reverse dependence of the compressibility on the density, clearly observed for polymorphs α, β, and ε (Figure 1a). These effects and the magnitudes of compressibility measured for resorcinol are typical of molecular crystals with hydrogen bonds. For example, in imidazole, at 0.1 MPa/296 K, β_v_ is 0.15 GPa^–1^, but it decreases to 0.07 GPa^–1^ at 0.7 GPa.^45^ In benzimidazole at 0.1 MPa the compressibility is similar, about 0.13 GPa^–1^, for polymorphs α and β, but the magnitude of β_v_ at 0.7 GPa in polymorph β decreases to 0.07 GPa^–1^.^18^

### Structural Model of Internal Pressure

**Figure 4:**
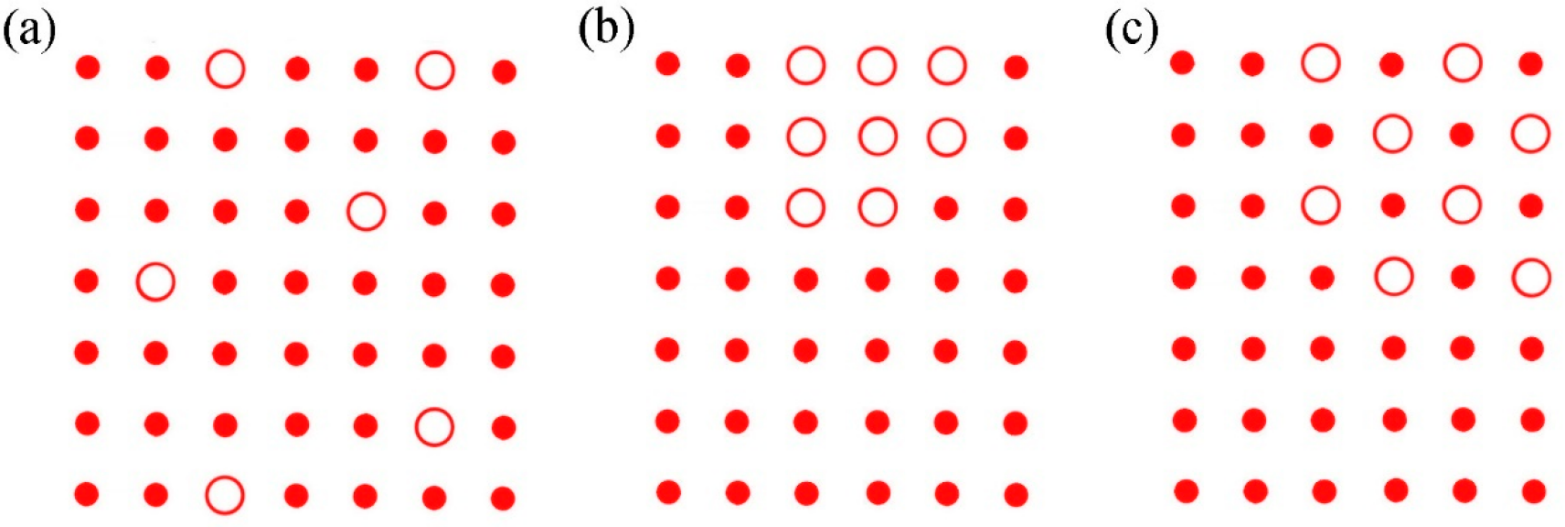
Schematic illustration of (a) a random distribution of single dopant molecules (large open circles), (b) dopant inclusion, and (c) dopant–host cocrystal inclusion in the host lattice (small dots).

The formation of polymorph ε in the frozen melt of resorcinol mixed with d-Ta suggested that mixing produces a similar effect as compression. Below, dopant molecules are indeed shown to be able to induce internal strain mimicking external compression. The generation of the internal doping pressure (*p*_d_) requires that the dopant molecule be embedded in the host structure, in which the host lattice is preserved (Figure 4a), and that no inclusions of dopant aggregates are formed (Figure 4, parts b and c). In the structure where the isolated dopant molecules are on average separated by *n* host molecules, the dopant-to-host molar ratio (*c*_d_) is
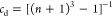
1

According to this formula, the average separation *n* = 1 implies a molar ratio equal to 1:7 (14.3% dopant), *n* = 2 gives a ratio of 1:26 (3.8%), *n* = 3 gives a ratio of 1:80 (1.25%), etc. For large *n* values, *c*_d_ can be approximated by the molar concentration of the dopant in the mixture, equal to (*n* + 1)^−3^. Assuming that the isotropic host and dopant molecules have a regular structure, the internal doping pressure can be assessed by the following formula:
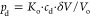
2where *K*_o_ is the bulk modulus of the pure host compound, δ*V* is the difference between the molecular volumes of the dopant and host, δ*V* = (*V*_d_ – *V*_o_), and *V*_o_ is the molecular volume of the pure host compound. Bulk modulus *K*_o_ can be substituted with volume compressibility (*K*_o_ = 1/β_v_), and eq 2 can be rewritten in the form *p*_d_ = (1/β_v_)*c*_d_δ*V*/*V*_o_.

Equation 2 has been derived with the assumptions of isolated dopant molecules embedded in the host lattice, the same bulk modulus (*K*_o_) describing the compression of the pure host and the doped crystal, and a spherical shape and isotopic interactions of all molecules. Thus, the effects of mismatched directional interactions, conformational flexibility, etc. are neglected. The obtained type of solid mixture (Figure 4) strongly depends on the method of crystallization and on the specific properties of the two compounds. For most compounds, the aggregation or cocrystallization of the dopant (Figure 4, parts b and c) can be expected to be favored by dynamic (slow) crystallization, while a random distribution of isolated dopant molecules in the host lattice (Figure 4a) is more likely to occur for kinetic processes, such as quick quenching of molten mixtures. Several different types of aggregation appear to be able to proceed simultaneously, but they can be dominated by the nucleation process. In quick and kinetic quenching of the melt, some parts of the components can also form films or amorphize, which is difficult to detect by PXRD. When several types of solidification occur in the frozen melt, the actual concentration of isolated dopant molecules in the obtained solid solution is smaller than the ratio of mixed components. In fact, this effect is the main principle behind the zone-melting method. These considerations alone show that milling is very unlikely to result in a uniform distribution of single dopant molecules in the single-crystal grain (Figure S10). In practice, the dopant compound must be torn into single molecules and the host crystals cleaved along every second or third layer. Melting is much more suitable for this purpose, which has been fully confirmed by the positive results of our melting-and-freezing experiments and negative results of our milling experiments on mixing the various dopant and host compounds presented below.

**Figure 5:**
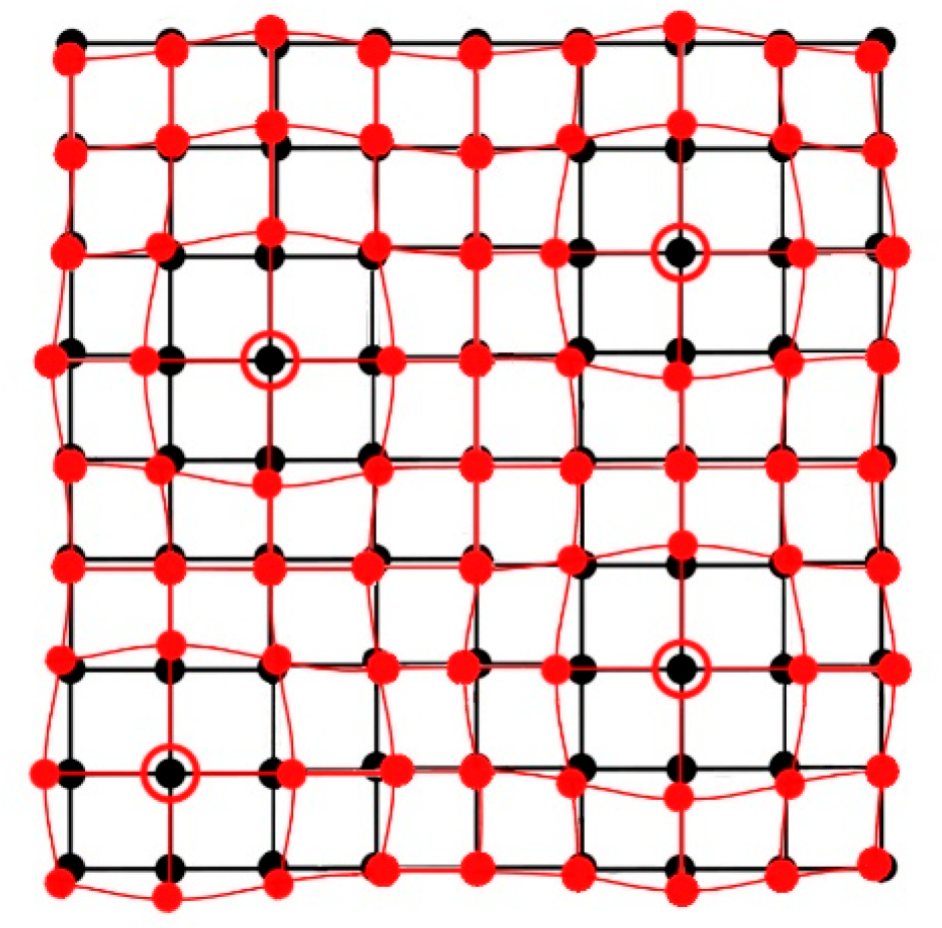
Schematic illustration of the effect of crystal doping (large open circles represent dopant molecules) on the crystal lattice: the lattice of a pure compound is represented by black lines, whereas red dots and lines represent the lattice strained under the internal pressure generated by large dopant molecules.

In the real liquid solution of the dopant and host molten mixture, the larger dopant molecules exert some pressure. Likewise, in the crystal, the structure around larger dopant molecules is squeezed (Figure 5). This internal pressure mimics the effect of external compression. Consequently, such doping of a compound can favor nucleation of high-pressure phases and their stabilization under ambient conditions. Figure 5 schematically illustrates that the microscopic strain induced by doping can be quite inhomogeneous, which can lead to a variety of nuclei and mixed phases in the kinetically frozen mixture. The double role of doping, i.e., the favored nucleation of the high-pressure polymorph and the stabilization of this polymorph by the dopant centers, can be explained by the internal pressure around the dopant molecules.

As explained above, the most efficient generation of strain is achieved when the dopant molecules do not form clusters in the structure of host compound. It is apparent that the equilibria between the intermolecular interactions of host–host, host–dopant, and dopant–dopant are essential for the homogeneous distribution of the dopant molecules in the host structure. Thus, apart from the molecular volume, also the compatibility of intermolecular forces types in the interactions, such as hydrogen bonds, van der Waals interactions, or halogen bonds, should be considered as the microscopic properties of the host and dopant compounds, or their hydrophilicity, lipophilicity, and miscibility as the macroscopic properties. Besides, the method of mixing the compounds may be crucial for obtaining the high dispersion of dopant molecules in the host structure, e.g., quick freezing from the melt, milling types, etc.

### High-Pressure Polymorphs under Ambient Conditions

**Scheme 1:**
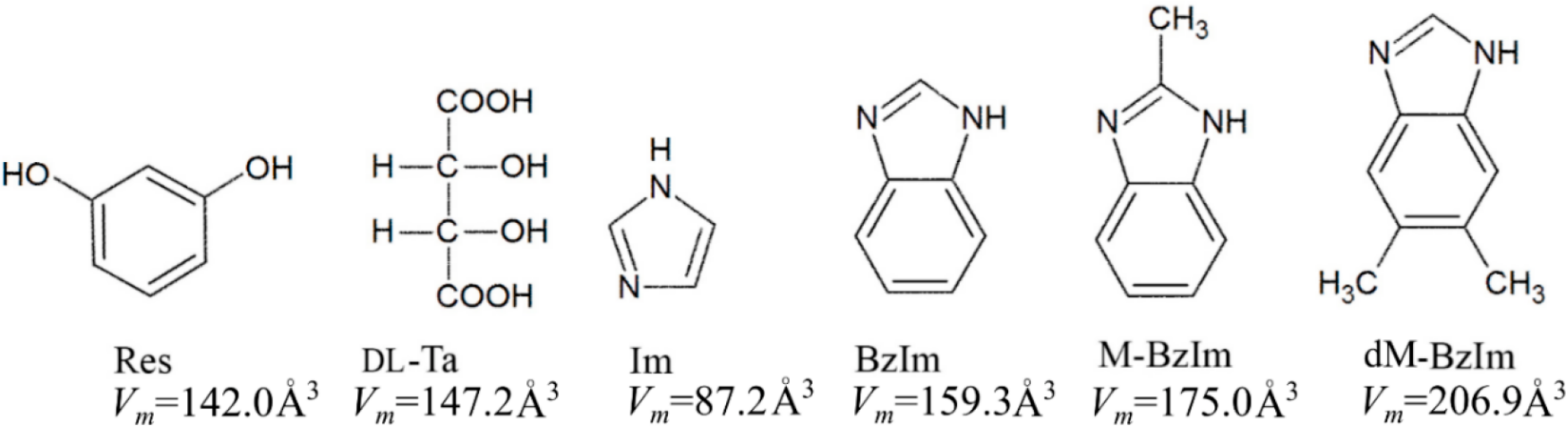
Molecular Formulas, Abbreviations, and Molecular Volumes, *V*_m_, of Compounds Used for Our Doping Tests at Ambient Pressure The *V*_m_ values of these compounds have been calculated according to their crystal data (refs (27 and 45−50)).

We tested the concept of doping pressure for crystallization of resorcinol and several imidazole derivates, for which high-pressure phases were previously reported.^18,20,45^ The formulas of these compounds and their molecular volumes are shown in Scheme 1.

The dopant compounds were chosen according to their molecular volume, larger than that of the host compound. The volume of Ta molecules is only approximately 5% larger than that of resorcinol; however, the mismatched interactions with the crystal environment can cause additional strain. The results of quantitative PXRD on the frozen resorcinol–dopant mixtures are summarized in Table 3 and Table S6. The polymorphs were identified by comparing the measured PXRD patterns with those generated for the crystal structures of the polymorphs, and the quantities of the components were calculated from the intensities of reflections (Figures S11–S26). The 15 wt % addition of dl-Ta to resorcinol yielded the largest amount of polymorph ε (Table 3) mixed with polymorph β. In accordance with Ostwald’s rule of stages, the presence of polymorph β is expected due to its stability temperature being below the melting point at 0.1 MPa. The internal pressures calculated according to eq 2 are given in Table 3 (Tables S4–S6).

**Table 3:** Polymorphs of Resorcinol Obtained in Molten and Frozen Resorcinol/Dopant Mixtures

dopant (wt %)	*c*_d_	dopant pressure *p*_d_ (GPa)	resorcinol polymorphs
l-Ta 15%	0.1295	0.52	15% β:85% ε
d-Ta 15%	0.1295	0.52	12% β:88% ε
dl-Ta 15%	0.1295	0.52	10% β:90% ε
M-BzIm 15%	0.1470	0.42	77% β:23% ε
M-BzIm 25%	0.2777	0.79	31% β:69% ε
dM-BzIm 25%	0.2509	1.26	31% β:69% ε

Analogous experiments were conducted for imidazole and its derivatives (Table 4 and Table S6), for which the ambient- and high-pressure polymorphs were previously determined.^18,20,45^ They showed results similar to those obtained for the doping experiments with resorcinol in that, in most cases, the high-pressure phases prevailed for benzimidazole (BzIm): the space-group symmetry of ambient-pressure polymorph α is *Pna*2_1_,^47^ and that of polymorph β stable above 0.23 GPa is *Pccn*.^18^ In the experiment of mixing BzIm with 5% dM-BzIm, we obtained a 100% yield of doped BzIm polymorph β. This was the only case of full yield of the high-pressure polymorph in all our experiments. This doped β-BzIm sample was stable for at least several months. The series of experiments on doping 2-methlybenimidazole (M-BzIm) with 5,6-dimethibenzimidazole (dM-BzIm) gave in the case of M-BzIm mixed with 5% dM-BzIm only the ambient-pressure phase α-M-BzIm, which was consistent with the internal pressure *p*_d_ (cf. eq 2, Table 4) being lower than the pressure of 0.26 GPa^20^ required for stabilizing pure polymorph β-M-BzIm. However, 15% and 25% doping caused the formation of the β-M-BzIm polymorph as the main component of the frozen mixture (Table 4).

**Table 4:** Polymorphs of Imidazole, Benzimidazole (BzIm), and 2-Methylbenzimidazole (M-BzIm) Doped with the Compounds Listed in Table 2 (cf. Scheme 1) at 5, 15, and 25 wt % Ratios

dopant (wt %)	*c*_d_	dopant pressure *p*_d_ (GPa)	Im polymorphs
BzIm 5%	0.0303	0.17	100% α
BzIm 25%	0.1920	1.11	35% α:65% β
M-BzIm 25%	0.1716	1.34	64% α:36% β

dopant (wt %)	*c*_d_	dopant pressure *p*_d_ (GPa)	BzIm polymorphs
M-BzIm 5%	0.0469	0.07	100% β
M-BzIm 15%	0.1588	0.22	5% α:95% β
dM-BzIm 15%	0.1436	0.44	13% α:87% β

dopant (wt %)	*c*_d_	dopant pressure *p*_d_ (GPa)	M-BzIm polymorphs
dM-BzIm 5%	0.0475	0.10	100% α
dM-BzIm 15%	0.1606	0.36	23% α:77% β
dM-BzIm 25%	0.3012	0.67	18% α:82% β

The absence of the higher-pressure polymorph ζ in any of the doped samples shows that sufficiently high internal strain cannot be generated by increasing the dopant concentration alone. It appears that when the dopant concentration exceeds some value (which may be different for different host and dopant compounds) the pressure does not increase linearly as a function of *c*_d_, as suggested by eq 2. This can be expected, because the regions of the host structure decrease when *c*_d_ increases and there are fewer single-dopant centers, which are most efficient in exerting strain on the host structure environment, while there are more larger clusters of the dopant molecules, less efficient in generating strain. Second, the higher-pressure polymorph (ζ) may require a close crystal packing possible only for tightly fitting identical molecules. It is also possible that Ostwald’s rule of stages remains valid for the nucleation of polymorphs in the doped-sample conditions, even when the internal strain exceeds that required for a new phase. At present, more experimental information is required to better understand the doping and high-pressure polymorphism.

## Conclusions

Rational doping by molecules larger than the host compound molecules can generate an internal strain in the melt, which mimics external compression and leads to high-pressure polymorphs. The doping pressure concept requires that single dopant molecules larger than the host molecules be randomly distributed in the host melt and crystal structure. Then, the internal pressure can be assessed from the molecular volume difference, dopant concentration, and host bulk modulus. The doping pressure is strongly inhomogeneous, but it shifts the thermodynamic equilibrium for nucleation toward high-pressure polymorphs and stabilizes them under ambient conditions. The optimized doping agent, its concentration, and the conditions when freezing the molten mixture can lead to a high or even full yield of desired high-pressure polymorphs. Undoubtedly, rational doping provides access to high-pressure polymorphs under ambient conditions and to their practical applications, for example, as APIs. The dopant properties can be adjusted for the envisaged applications of the product; for example, they may be chosen to improve the taste or bioaccessibility of the API. The high-pressure polymorphs of resorcinol appear much less puzzling than the polymorphs in varied-temperature studies alone. In the sequence of high-pressure phases, their increased density is obligatory, while their symmetry changes (Table 1) are not generally required, although they occur in most of the first-order phase transitions. These relations between resorcinol polymorphs α and β as a function of temperature, the unexpected density change, and the same space-group symmetry type appeared particularly confusing in the 1930s, when resorcinol happened to be the first example of structurally characterized polymorphs of any organic compound.^27,28^ The extended hysteresis of the transition between phases α and β only complicated the description. For these reasons, resorcinol was excluded from some of the most prominent reviews and textbooks on polymorphism. Presently, the case of resorcinol can be regarded as a clear illustration of the necessary and sufficient conditions in thermodynamics.^51^

## Supporting Information Available

Crystallographic data, powder diffraction data, and the internal dopant pressure calculation (PDF)

## Notes

The X-ray crystallographic structures reported in this study have been deposited at the Cambridge Crystallographic Data Centre under deposition numbers CSD 2084039–2084046. These data can be obtained free of charge from https://www.ccdc.cam.ac.uk/.
