# Supplementary material for: High-Pressure Polymorphs Nucleated and Stabilized by Rational Doping under Ambient Conditions
Source: J Phys Chem C Nanomater Interfaces. 2021 Oct 19;125(42):23501–9. doi: 10.1021/acs.jpcc.1c07297 (PMC8559611; doi:10.1021/acs.jpcc.1c07297)
Supplement: Supplementary file 1 — jp1c07297_si_001.pdf [file jp1c07297_si_001.pdf]

Supporting Information

High-pressure Polymorphs Nucleated and Stabilized by Rational Doping under Ambient Conditions

*Fatemeh Safari,   Andrzej Katrusiak**

*Email: katran@amu.edu.pl

Faculty of Chemistry, Department of Materials Chemistry, Adam Mickiewicz University, ul Uniwersytetu Poznańskiego 8, 61-614 Poznań, Poland

Index

Table of Contents

Index **2**

1 High-Pressure Crystallizations **3**

1.1 Crystallization of resorcinol polymorphs 3

1.2 Crystallographic data 4

1.3 Influence of pressure on unit-cell parameters & geometry 6

1.4 Molecular packing 8

2 Powder X-ray Diffraction (PXRD) Data **9**

2.1 Optical micrographs of resorcinol 9

2.2 Milling mixed samples 9

2.3 2D diffraction patterns of pure compounds 10

2.4 2D Diffraction patterns at the present of dopant 13

3 Internal Dopant Pressure **18**

3.1 Molecular volume (V_m) 18

3.2 Van der Waals volume (V_{Waals}) 18

3.3 Isothermal compressibility 18

1 High-Pressure Crystallizations

1.1 Crystallization of resorcinol polymorphs

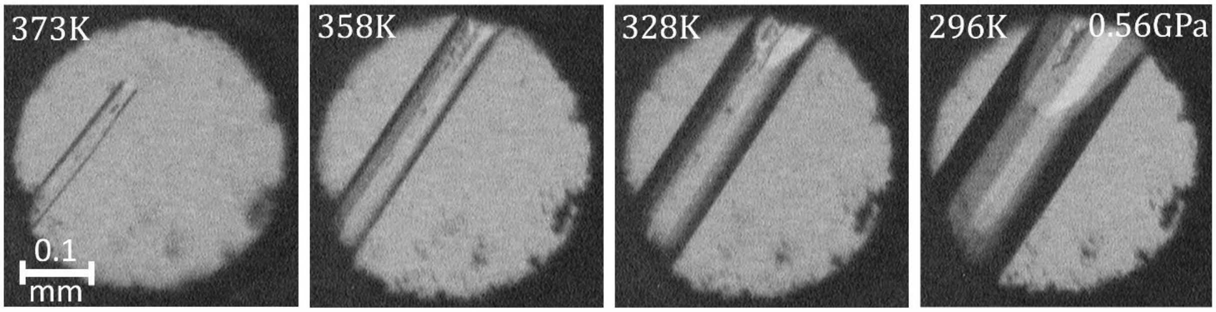

Figure S1| Isochoric crystallization of polymorph ε from the solution of pure resorcinol in the 50:50 methanol: water mixture. Several ruby chips for pressure calibration lie along the right side of the chamber.

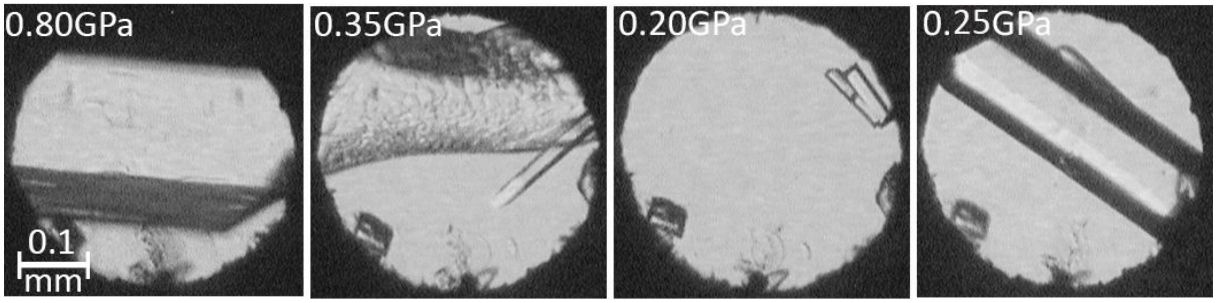

Figure S2| Single crystal of polymorph ε obtained of the monohydrate crystal isothermally dissolved in water by releasing pressure up to 0.20 GPa and then increasing it to 0.25 GPa. Polymorph ε nucleated at 0.35 GPa, but at 0.20 GPa it changed its shape and continued to grow isothermally up to 0.25 GPa. Three small ruby chips lie at the bottom edge of the gasket.

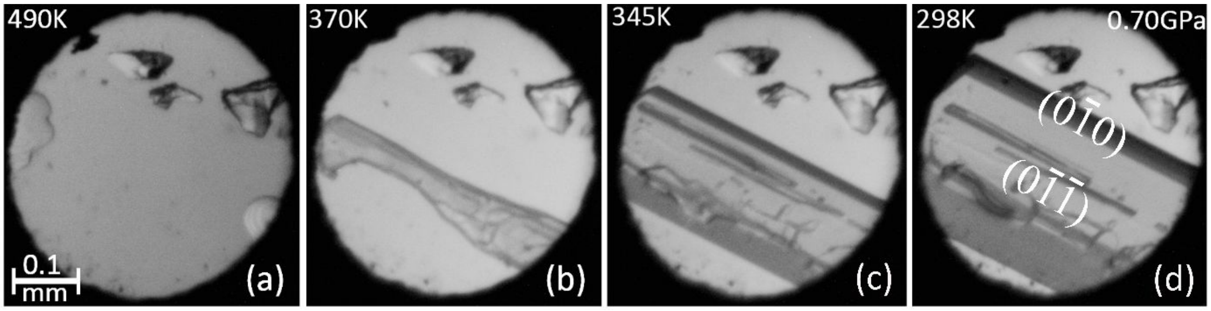

Figure S3│ Polymorph ζ nucleated in isochoric conditions from the H2O: MeOH: EtOH (1:16:3 vol.) mixture: (a) at 490 K, (b) 370 K, (c) 345 K and (d) the crystal with sharp edges and its final morphology at 298 K. Several tiny ruby chips for the pressure calibration lie above the crystal.

1.2 Crystallographic data

Table S1│ Selected crystal data of ε -polymorph of resorcinol.

$C_6H_6O_2$	Phase ε	Phase ε	Phase ε	Phase ε
Pressure (GPa)	0.25(2)	0.56(2)	0.68(2)	0.96(2)
Temperature (K)	296(2)			
Formula weight	110.11			
Crystal color	colorless			
Crystal size (mm)	0.35×0.12×0.1	0.35×0.12×0.1	0.34×0.15×0.1	0.34×0.15×0.1
Crystal system	Orthorhombic			
Space group	$P2_12_12_1$			
Unit cell (Å) *a*	17.876(5)	17.787(4)	17.789(2)	17.700(6)
b	10.464(6)	10.273(2)	10.2289(11)	10.094(3)
c	5.7045(16)	5.6550(4)	5.65644(17)	5.6096(6)
Volume (Å^3)	1067.0(8)	1033.3(3)	1029.25(17)	1002.2(5)
Z/*Z'*	8/2	8/2	8/2	8/2
Density (g/cm^3)	1.371	1.416	1.421	1.459
Wavelength MoKα (Å)	0.71073	0.71073	0.71073	0.71073

Absorption (mm^{-1})	0.103	0.107	0.107	0.110
F(000)	464.0	464	464	464
2θ max (°)	53.136	54.304	53.106	54.088
Index ranges h_{min}/h_{max}	-19/19	-15/15	-15/15	-16/16
k_{min}/k_{max}	-6/6	-9/9	-9/9	-9/9
l_{min}/l_{max}	-7/7	-7/7	-7/7	-7/7
Refl. Collected	3856	4361	3857	4197
Refl. observed (I>4σ_I)	819	747	687	909
R(int)	0.1142	0.0987	0.0386	0.0833
Data/restraints/parameters	827/18/156	751/14/155	687/0/145	909/6/145
Goodness-of-fit on F^2	1.047	1.055	1.126	1.016
Final R_1 (I>2σ_I)	0.0760/0.1808	0.0425/0.0738	0.0298/0.0623	0.0480/0.0722
R_1/ wR_2 (all data)	0.1604/0.2288	0.1014/0.0931	0.0434/0.0665	0.1091/0.0909
Absorption corrections	DAC, gasket and sample crystal			

Table S2│ Selected crystal data of ζ -polymorph of resorcinol.

$C_6H_6O_2$	Phase ζ	Phase ζ	Phase ζ	Phase ζ
Pressure (GPa)	0.70(2)	0.83(2)	1.0(2)	1.20(2)
Temperature (K)	296(2)			
Formula weight	110.11			
Crystal color	colorless			
Crystal size (mm)	0.30×0.12×0.1	0.30×0.12×0.1	0.30×0.12×0.1	0.30×0.12×0.1
Crystal system	Monoclinic			

Space group		$P2_1/c$		
Unit cell (Å) a	10.6348(8)	10.6318(18)	10.562(10)	10.465(3)
b	9.5004(16)	9.502(4)	9.47(2)	9.425(5)
c	10.873(2)	10.869(5)	10.83(2)	10.787(6)
β (°)	114.713(15)	114.88(3)	114.52(17)	113.70(4)
Volume (Å^3)	997.9(3)	996.1(6)	986 (3)	974.1(9)
Z/Z'	8/2	8/2	8/2	8/2
Density (g/cm^3)	1.466	1.468	1.484	1.5029
Wavelength MoKα (Å)	0.71073	0.71073	0.71073	0.71073
Absorption (mm^{-1})	0.110	0.111	0.112	0.113
F(000)	464.0	464	464	464
2θ max (°)	52.84	54.076	52.33	52.668
Index ranges h_{min}/h_{max}	-13/13	-13/13	-13/13	-13/12
k_{min}/k_{max}	-8/8	-8/8	-8/8	-8/7
l_{min}/l_{max}	-10/10	-11/10	-10/10	-10/10
Refl. Collected	3678	4061	2708	3516
Refl. observed (I>4σ$_I$)	610	625	568	593
R(int)	0.0352	0.0352	0.1289	0.0499
Data/restraints/parameters	610/0/149	625/0/149	568/12/147	593/6/149
Goodness-of-fit on F^2	1.057	1.150	1.018	1.144
Final R_1 (I>2σ$_I$)	0.0322/0.0749	0.0336/0.0763	0.0822/0.1679	0.0354/0.0736
R_1/wR_2 (all data)	0.0541/0.0861	0.0528/0.0859	0.1950/0.2334	0.0963/0.0942
Absorption corrections		DAC, gasket and sample crystal		

1.3 Influence of pressure on unit-cell parameters & geometry

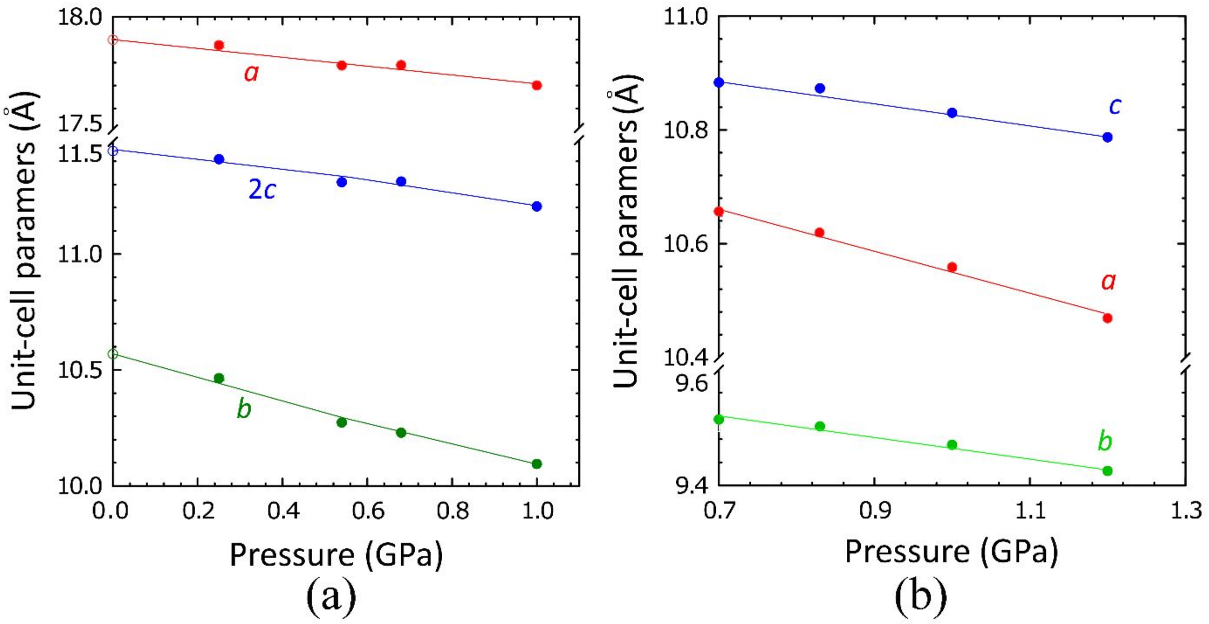

Figure S4| Unit -cell parameter as function of pressure for (a) ε-polymorph up to 1.0 GPa and (b) ζ-polymorph up to 1.2 GPa. The ESD is smaller than symbol in both plots. The empty symbols are determined for the ε phase obtained as a mixture of resorcinol and tartaric acid are taken from Zhu. *et al.*[1]

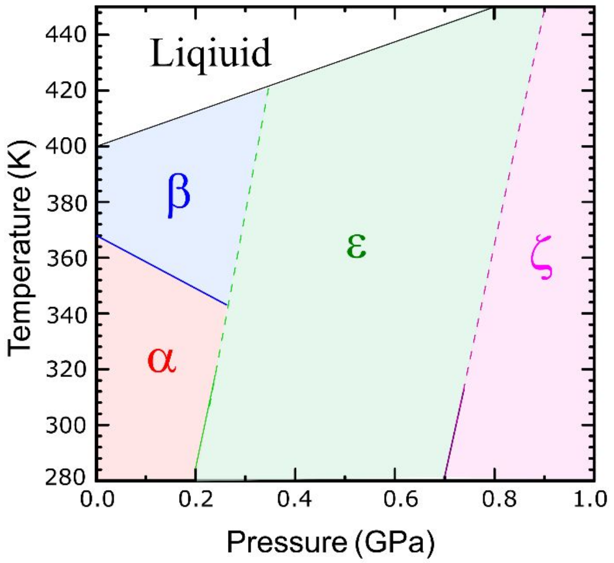

Figure S5| *p–T* diagram of resorcinol phases (labeled by Greek letters) according to this study. The dashed lines indicate the extrapolated boundaries, beyond those determined at 296 K in this study. The phase boundaries were determined in high-pressure recrystallization experiments.

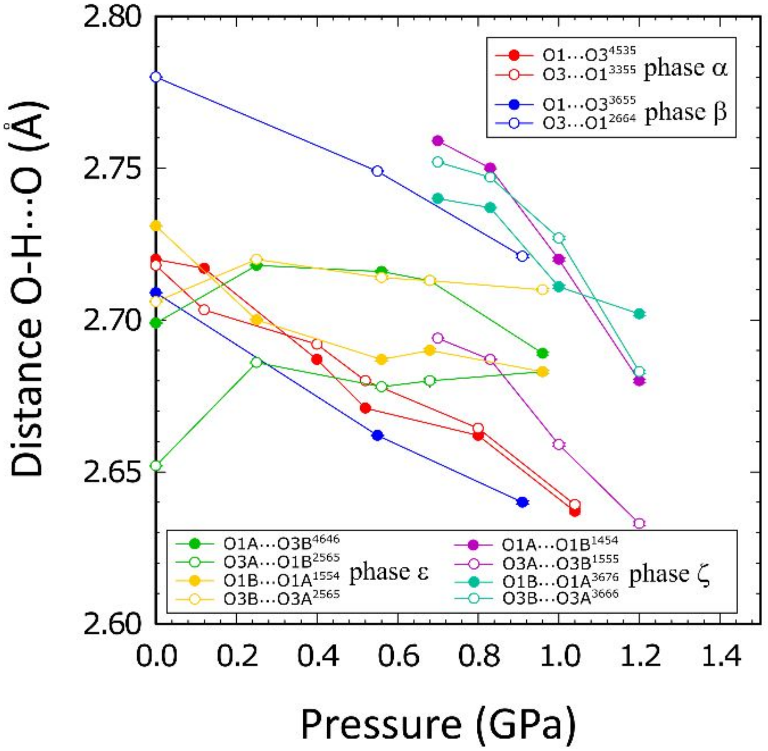

Figure S6| Pressure dependence of hydrogen-bond distances O···O with the symmetry codes of each neighbor molecules.

Table S3| The ORTEP codes and their explicit forms of symmetry transformations.[2]

Phase α	
ORTEP code	symmetry transformation
3355	-3/2+*x*,1/2+*y*, 1/2+*z*
4535	1/2+*x*,-3/2-*y*, +*z*
Phase β	
ORTEP code	symmetry transformation
2664	1-*x*, 1-*y*, -1/2+*z*
3655	3/2-*x*, 1/2+*y*, 1/2+*z*
Phase ε	
ORTEP code	symmetry transformation
1554	*x*, *y*, -1+*z*

2565	1/2-*x*, 1-*y*, 1/2+*z*
4646	1-*x*, -1/2+*y*, -3/2-*z*
Phase ζ	
ORTEP code	symmetry transformation
1555	*x*, *y*, *z*
1454	-1+*x*, +*y*, -1+*z*
3666	1-*x*, 1-*y*, 1-*z*
3676	1-*x*, 2-*y*, 1-*z*

1.4 Molecular packing

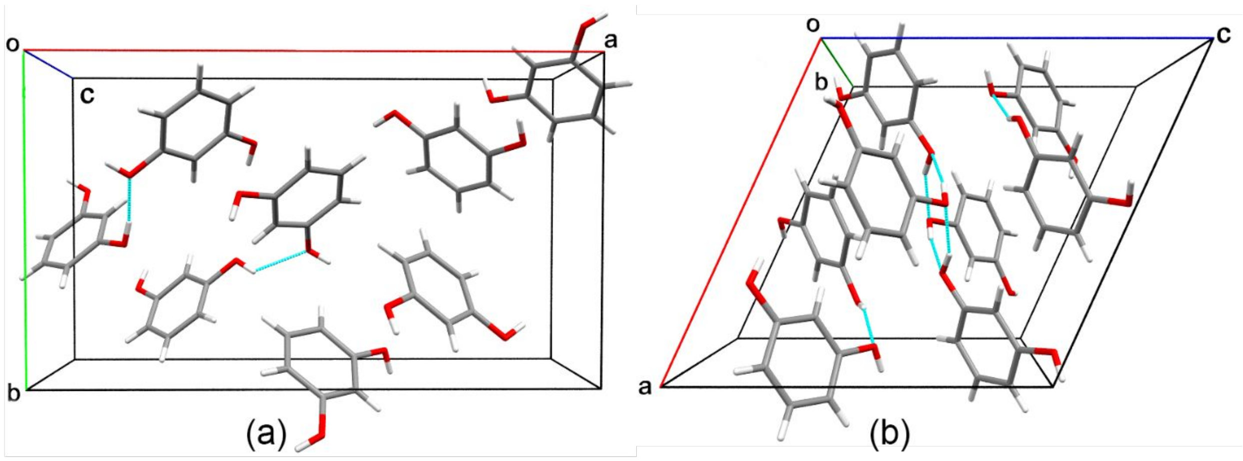

Figure S7 | Autostereogram[3] of the molecular packing in (a) ε- resorcinol structure at 0.25 GPa/296 K and (b) ζ-resorcinol at 0.70GPa/296K. The OH···O hydrogen bonds are indicated as cyan lines.

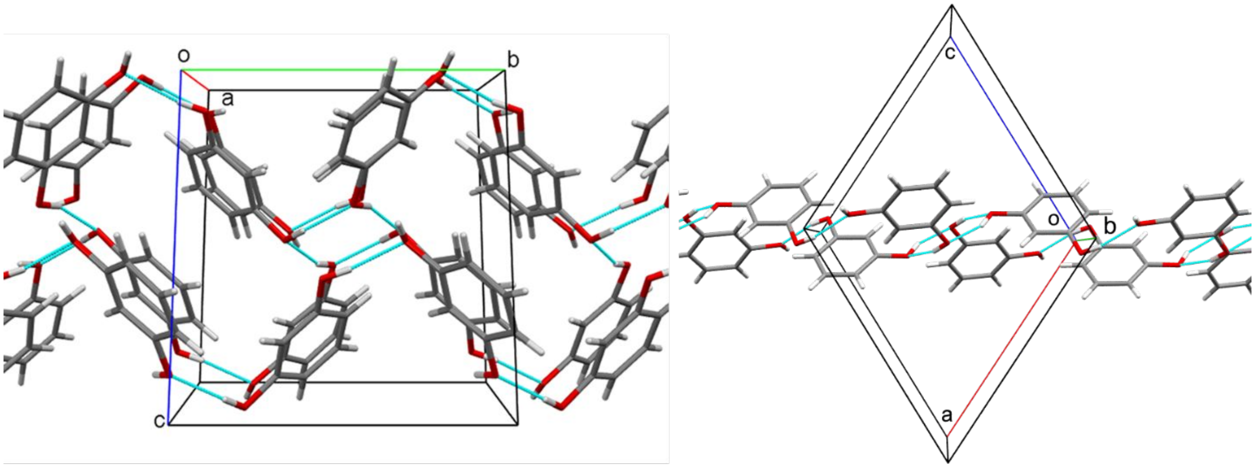

(a) (b)

Figure S8 | (a) Autostereogram[3] of molecular packing in ζ-polymorph at 0.7 GPa in the [100] direction. The hydrogen bonds are indicated as cyan line. (b) One layer of molecules viewed along the [010] direction.

2 Powder X-ray Diffraction (PXRD) Data

2.1 Optical micrographs of resorcinol

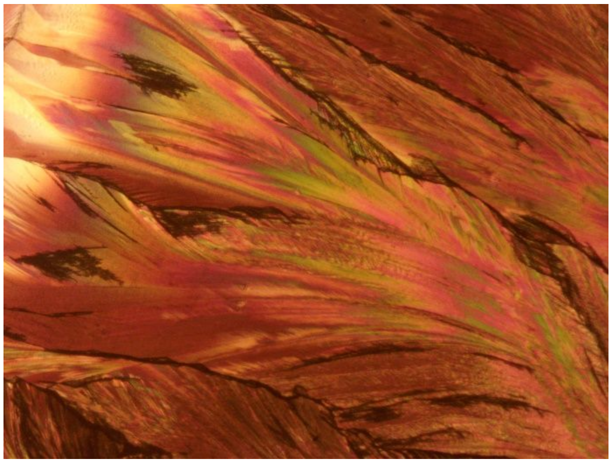

Figure S9 | Polarized-light optical micrographs of Res+0.15 wt% DL.Ta (ε form) at room temperature.

2.2 Milling mixed samples

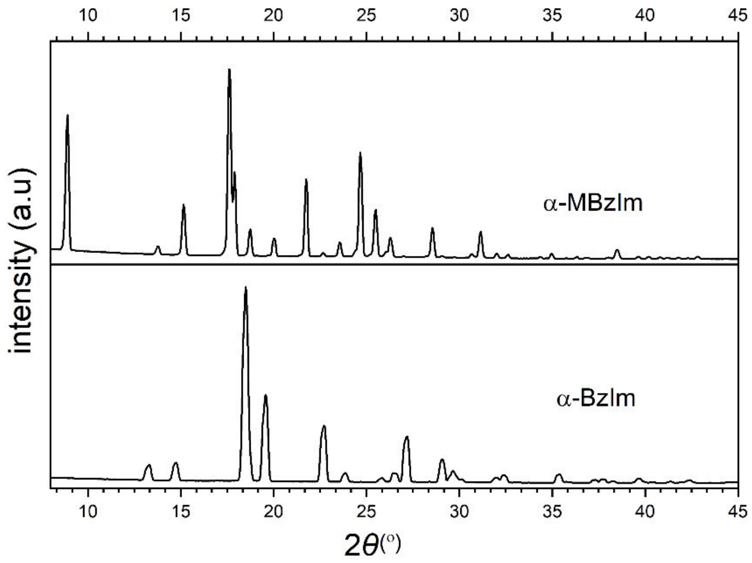

Figure S10 | Diffraction pattern of benzimidazole and 2-methylimidazole doped with 15 wt.% dM-BzIm after 4h milling.

2.3 2D diffraction patterns of pure compounds

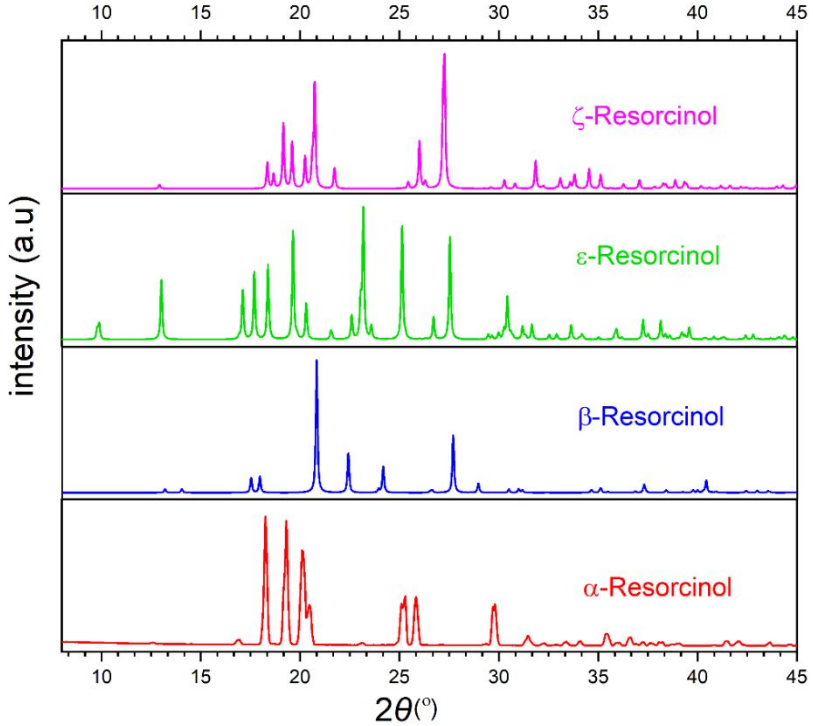

Figure S11│ Diffraction pattern of pure resorcinol α, β at ambient condition and polymorph ε at 0.25GPa, polymorphs ζ at 0.70GPa. The powder diffraction of high pressure generated from mercury software.[4]

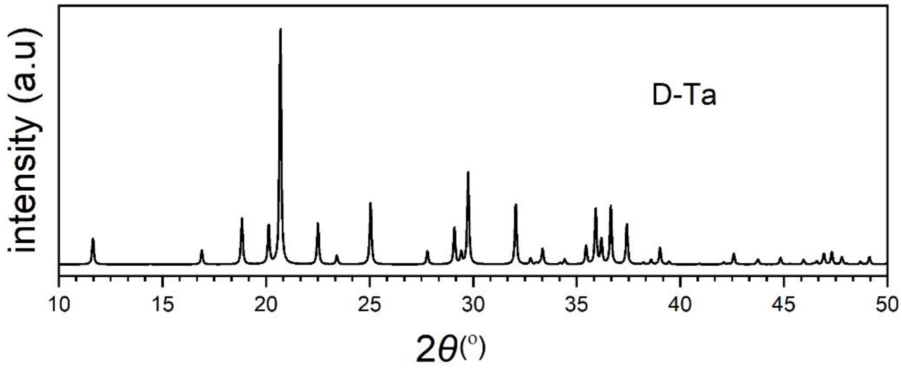

Figure S12│ Diffraction pattern of pure tartaric acid at 296K/0.1 MPa.[5]

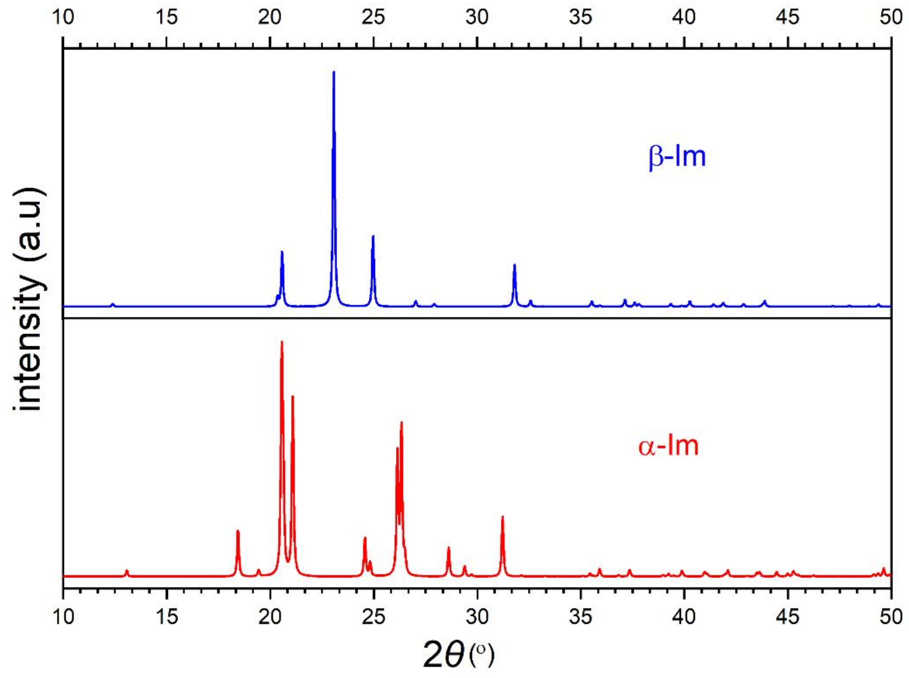

Figure S13│ Diffraction pattern of α-imidazole at ambient condition and β-imidazole at 0.5 GPa.[6,7]

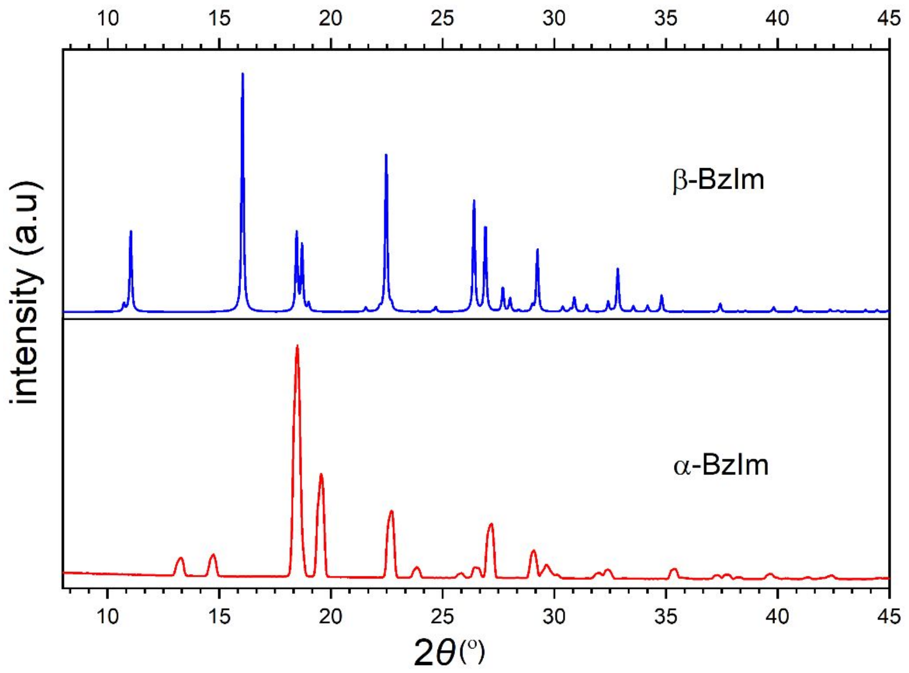

Figure S14│ Diffraction pattern of α-benzimidazole at ambient condition and β- benzimidazole at 0.23 GPa.[8,9]

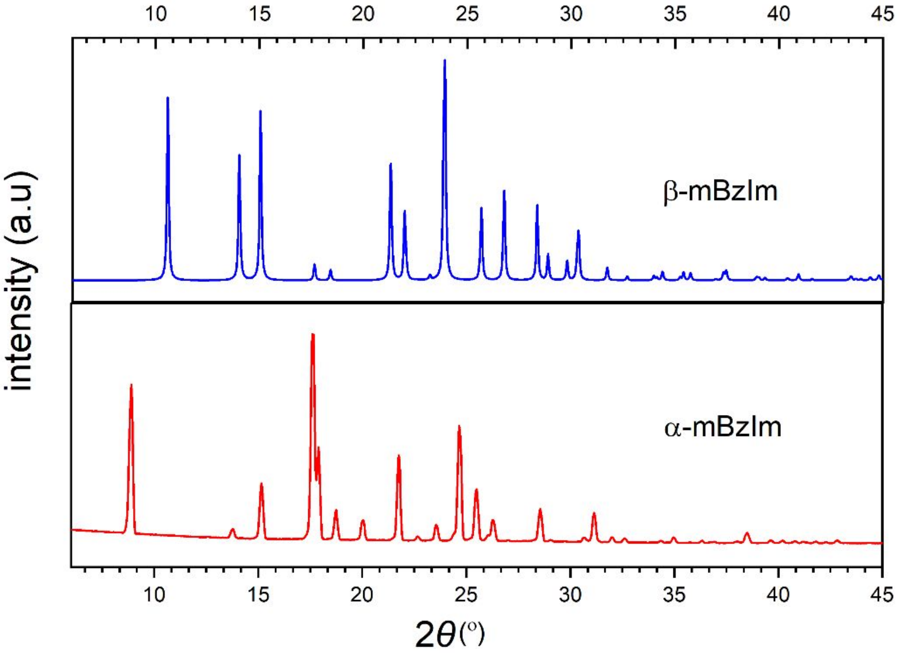

Figure S15│ Diffraction pattern of α-2-methyimidazol at ambient condition and β-2-methyimidazol at 0.26 GPa.[10,11]

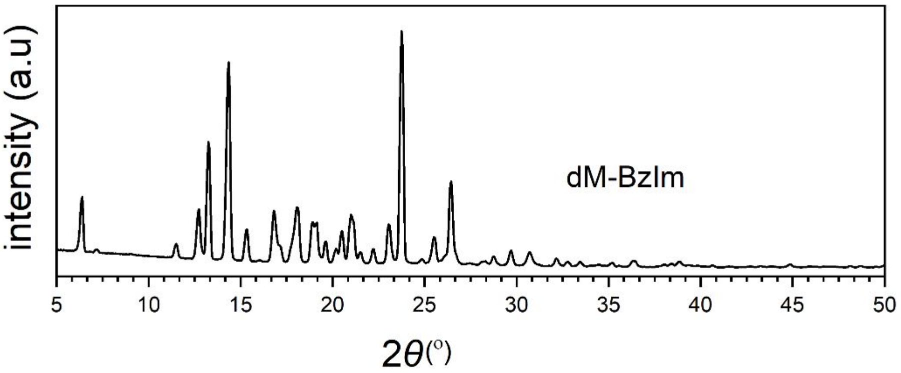

Figure S16│ Diffraction pattern of 5,6-dimethylbenzimidazole.at ambient condition.[12]

2.4 2D Diffraction patterns at the present of dopant

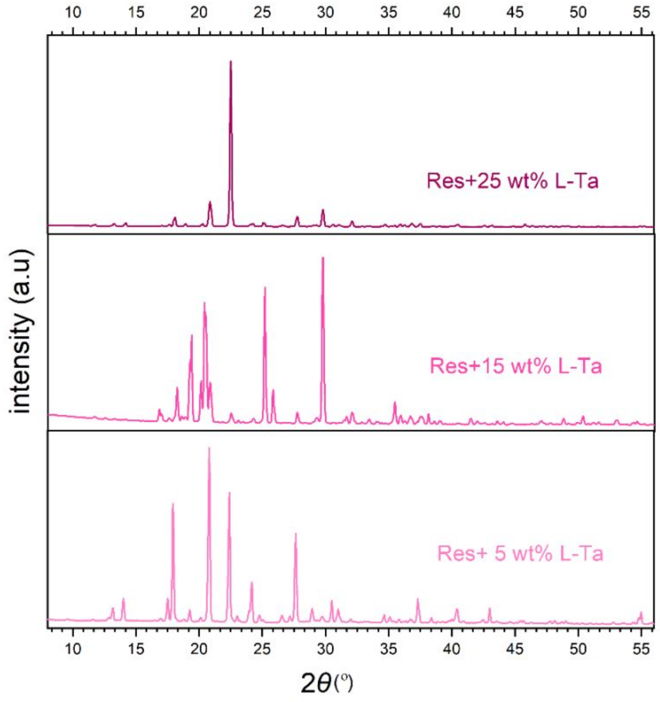

Figure S17| Diffraction pattern of resorcinol doped with 5-25 wt.% L-tartaric acid at ambient condition.

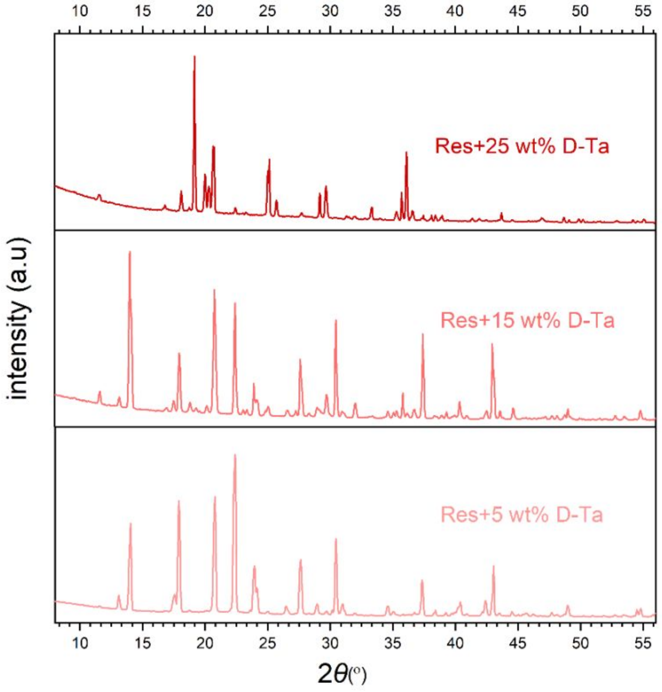

Figure S18| Diffraction patterns of resorcinol doped with 5-25 wt.% D-tartaric acid at ambient condition.

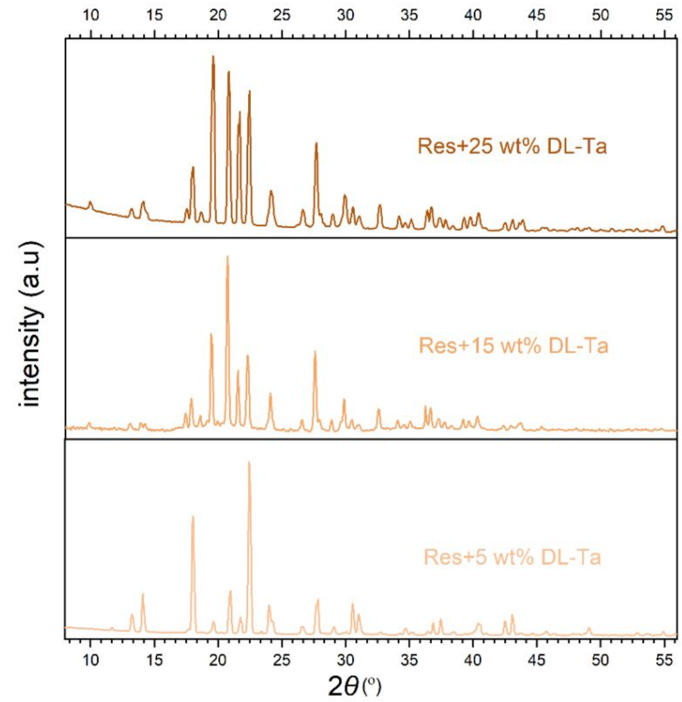

Figure S19│ Diffraction pattern of resorcinol doped with 5-25 wt.% DL-tartaric acid.at ambient condition.

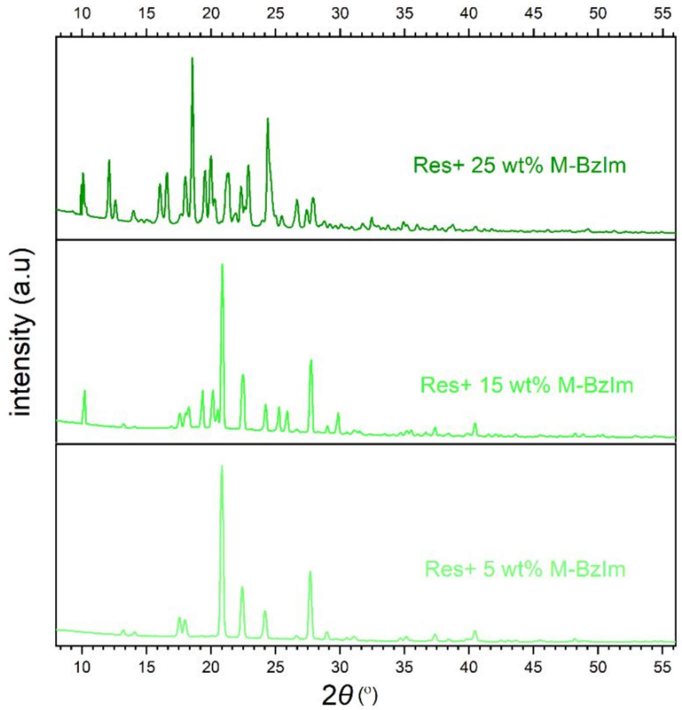

Figure S20│ Diffraction pattern of resorcinol doped with 5-25 wt.% 2-methyimidazol at ambient condition.

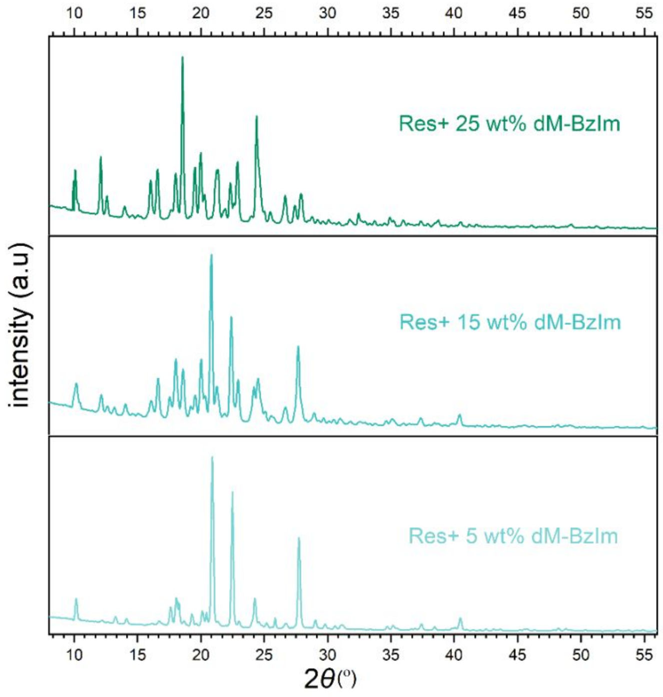

Figure S21│ Diffraction pattern of resorcinol doped with 5-25 wt.% 5,6-dimethylbenzimidazole at ambient condition.

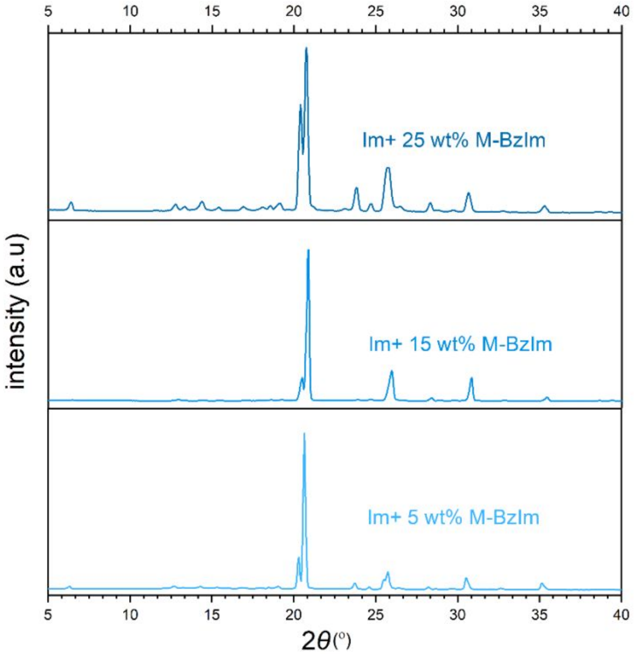

Figure S22│ Diffraction patterns of imidazole doped with 5-25 wt.% 2-methybenzimidazole.at ambient condition.

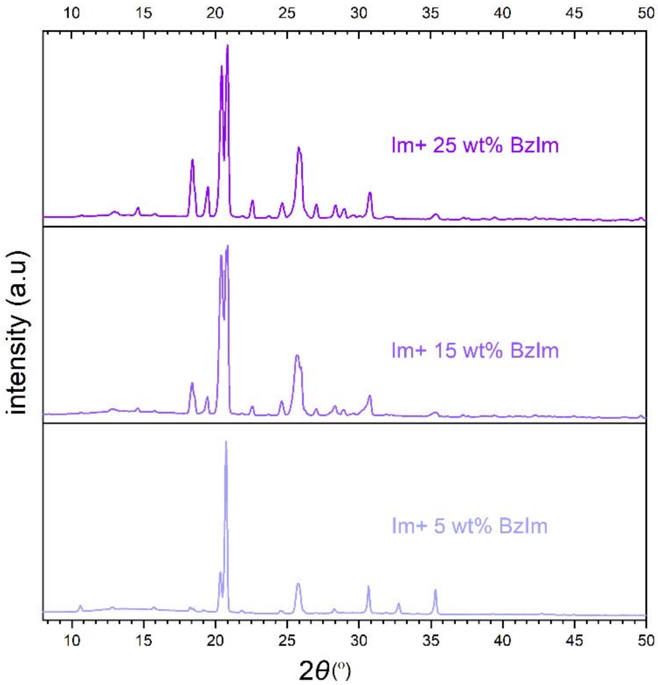

Figure S23│ Diffraction patterns of imidazole doped with 15 wt.% benzimidazole.at ambient condition.

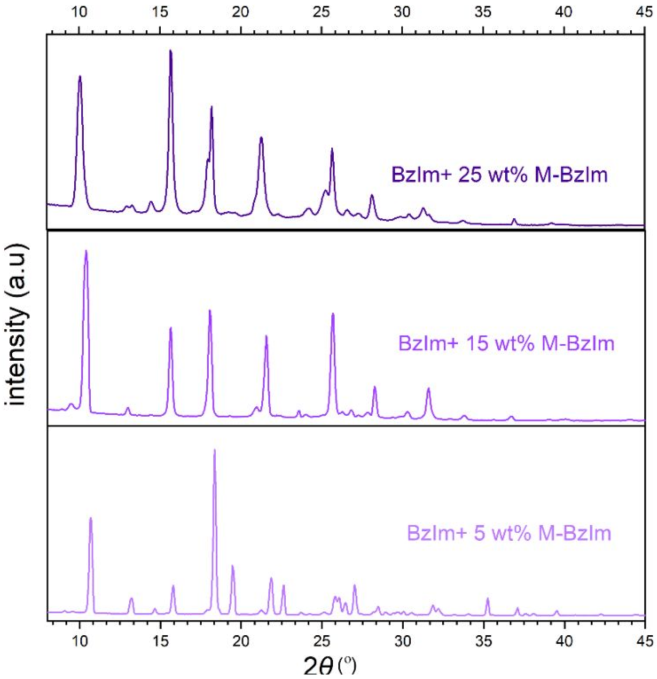

Figure S24| Diffraction patterns of benzimidazole doped with 5-25 wt.% 2-methylbenzimidazole.at ambient condition.

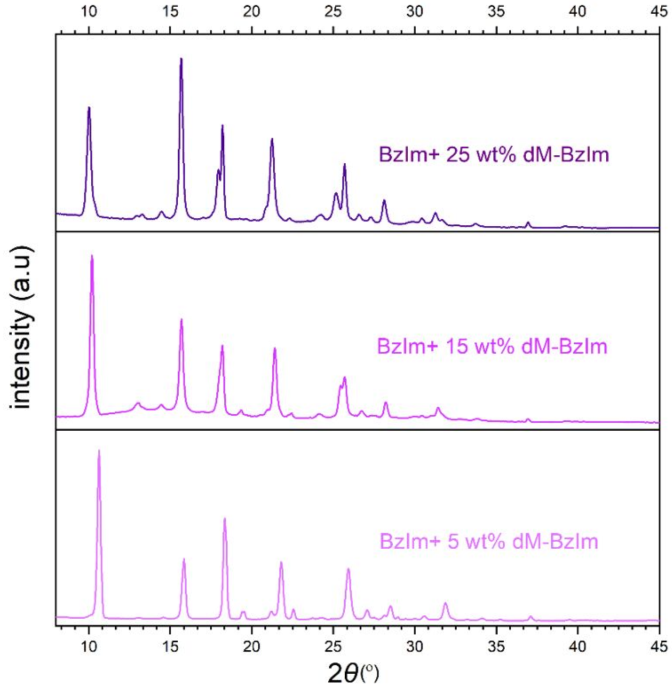

Figure S25| Diffraction patterns of benzimidazole doped with 5-25 wt.% 5,6-dimethylbenzimidazole.at ambient condition.

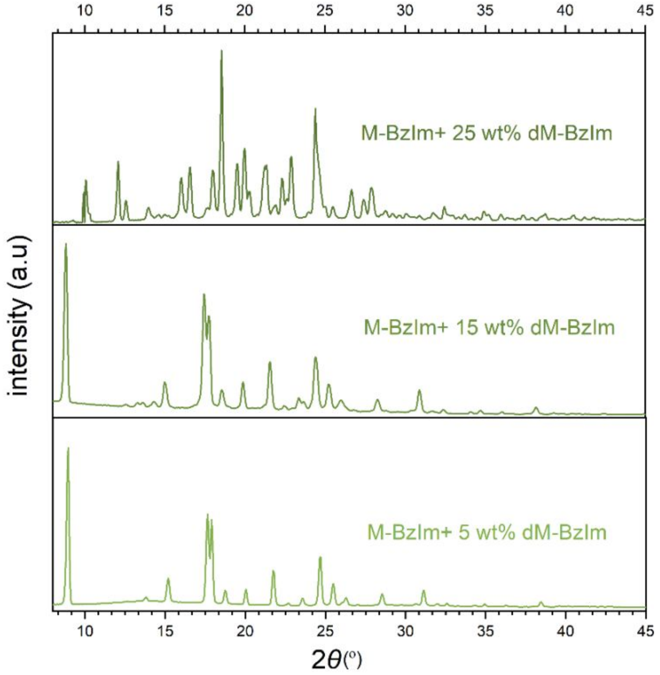

Figure S26| Diffraction patterns of 2-methylbenzimidazole doped with 5-25wt.% 5,6-dimethylbenzimidazole.

3 Internal Dopant Pressure

3.1 Molecular volume (V_m)

The molecular volume was calculated by divided crystal volume (*V*)on the number of formula units in the unit cell(*Z*) for each crystal structure.

3.2 Van der Waals volume (V_{Waals})

An ad-hock Fortran computer program for calculating the van der Waals volume of molecules, by summing micro voxels, was used. (Katrusuak, AMU, Poznan 2009). The program adds voxels contained within the van der Waals radii (after Bondi[13]) of all atoms in the molecule.

Table S4| The value of volume form single-crystal structures and molecules at ambient condition.

Compounds	V_m (Å^3)	V_{waals} (Å^3)
Imidazole	87.2	63.2
Resorcinol	142.0	95.1
Tartaric acid	147.2	112.0
Benzimidazole	159.3	104.8
2-methylbenzimidazole	175.0	118.7
5,6-dimethylbenzimidazole	206.9	135.4

3.3 Isothermal compressibility

Compressibility parameters β of volume V, calculated as βx=$-1x\partial x\partial p$, where x is V given value. The differential part was analytically calculated based on a quadratic polynomial fitted to the experimentally obtained pressure dependences of unit-cell volume V phase α of compounds.

Table S5│ Compressibility parameter β_V for high-pressure and ambient-pressure phases calculated based on the structural information obtained from the literature.[6-11]

V (p)	Compressibility	β_V[GPa^{-1}]
	High-pressure phases	
Im	$\beta_V = -\frac{1}{V0.5}(30.742p^2 - 105.33p + 663.1)$ (R= 0.9811)	0.11241
Res	$\beta_V = -\frac{1}{V0}(3.169p^2 - 91.96p + 1082.42)$ (R= 0.9939)	0.08469
BzIm	$\beta_V = -\frac{1}{V0}(17.19p^2 - 119.42p + 1234)$ (R= 0.9983)	0.09745
M- BzIm	$\beta_V = -\frac{1}{V0.26}(18.20p^2 - 95.23p + 1334.2)$ (R=0.9991)	0.06432
	Ambient-phases	
Im	$\beta_V = -\frac{1}{V0}(13.902p^2 - 64.916p + 348.7)$ (R= 0.9126)	0.18513
Res	$\beta_V = -\frac{1}{V0}(4.001p^2 - 48.674p + 567.91)$ (R= 0.9792)	0.08584
BzIm	$\beta_V = -\frac{1}{V0}(14.15p^2 - 70.126p + 637.5)$ (R= 0.9986)	0.10994
M- BzIm	$\beta_V = -\frac{1}{V0}(43.100p^2 - 86.31p + 1399.58)$ (R=0.9991)	0.06147

Table S6│ The value of internal dopant pressure by different wt% in Res as host with the percentage of high-pressure forms of each compound at room temperature.

Dopant	c_d	Dopant Pressure	Resorcinol
L-Ta 5%	0.0386	0.16	82% β :18% ε
L-Ta 15%	0.1295	0.52	15%β: 85% ε
L-Ta 25%	0.2445	0.97	90% β: 10%ε
D-Ta 5%	0.0386	0.16	100% β
D-Ta 15%	0.1295	0.52	12%β: 88% ε
D-Ta 25%	0.2445	0.97	90% β: 10%ε
DL-Ta 5%	0.0386	0.16	100% β
DL-Ta 15%	0.1295	0.52	10%β: 90% ε
DL-Ta 25%	0.2445	0.97	10%β: 90% ε
M-BzIm 5%	0.0438	0.12	100%β
M-BzIm 15%	0.1470	0.42	77% β: 23% ε

M-BzIm 25%	0.2777	0.79	31% β: 69% ε
dM- BzIm 5%	0.0396	0.16	83% β: 17% ε
dM- BzIm 15%	0.1336	0.67	78%β: 22% ε
dM- BzIm 25%	0.2509	1.26	31% β: 69% ε

Table S6│ (Continued). The value of internal dopant pressure by different wt% in Im, BzIm, M-BzIm as host with the percentage of high-pressure forms of each compound at room temperature.

Dopant	c_d	Dopant Pressure	Im
BzIm 5%	0.0303	0.17	100%α
BzIm 15%	0.1023	0.60	57%α :43%β
BzIm 25%	0.1920	1.11	35%α :65%β
M-BzIm 5%	0.0270	0.22	100%α
M-BzIm 15%	0.0915	0.72	79%α: 21%β
M-BzIm 25%	0.1716	1.34	64%α :36%β
Dopant	c_d	Dopant Pressure	BzIm
M-BzIm 5%	0.0469	0.07	100%β/
M-BzIm 15%	0.1588	0.22	5%α: 95%β
M-BzIm 25%	0.2977	0.42	13%α:87%β
dM- BzIm 5%	0.0425	0.14	100%β
dM- BzIm 15%	0.1436	0.44	13%α: 87%β
dM- BzIm 25%	0.2694	0.82	10%α:90%β
Dopant (wt %)	c_d	Dopant Pressure (GPa)	M-BzIm polymorphs
dM- BzIm 5%	0.0475	0.10	100%α
dM- BzIm 15%	0.1606	0.36	23%α:77% β
dM- BzIm 25%	0.3012	0.67	18%α: 82% β

References

(1) Zhu, Q.; Shtukenberg, A. G.; Carter, D. J.; Yang, T.; Yu, J.; Chen, M.; Raiteri, P.; Oganov, A. R.; Pokroy, B.; Polishchuk, I.; Bygrave, P. J.; Day, G. M.; Rohl, A. L.; Tuckerman, M. E.; Kahr, B. Resorcinol Crystallization from the Melt: A New Ambient Phase and New “Riddles. *J. Am. Chem. Soc.* **2016**, *138*, 4881−4889, DOI: 10.1021/jacs.6b01120

(2) Johnson C. K. *ORTEPII. Report ORNL-5138.* (Oak Ridge National Laboratory, Tennessee, USA, 1976)

(3) Katrusiak, A. Crystallographic Autostereograms. *J. Mol. Graphics Modell.* **2001**, *19*, 363– 367, DOI: 10.1016/S1093-3263(00)00085-1

(4) Macrae, C. F.; Bruno, I. J.; Chisholm, J. A.; Edgington, P. R.; McCabe, P.; Pidcock, E.; Rodriguez-Monge, L.; Taylor, R.; Van De Streek, J.; Wood, P. A. Mercury CSD 2.0 - New Features for the Visualization and Investigation of Crystal Structures. *J. Appl. Crystallogr.* **2008**, *41*, 466–470, DOI: 10.1107/S0021889807067908

(5) Bootsma, G. A.; Schoone. J. C. Crystal structures of mesotartaric acid. *Acta Cryst.* **1967**, *22*, 522–532, DOI: 10.1107/S0365110X67001070

(6) Martinez-Carrera, S. The crystal structure of imidazole at -150°. *Acta Crystallogr.* **1966**, *20*, 783–798, DOI: 10.1107/S0365110X66001853

(7) Paliwoda, D.; Dziubek, K. F.; Katrusiak, A. Imidazole hidden polar phase. *Cryst. Growth Des.* **2012**, *12*, 4302–4305, DOI: 10.1021/cg300852t

(8) Escande, A.; Galigné. J. L. Structure cristalline du benzimidazole, $C_7N_2H_6$: comparaison des résultats de deux études indépendantes. *Acta Cryst.* **1974**, *B30*, 1647–1648, DOI: 10.1107/S0567740874005528

(9) Zieliński, W.; Katrusiak, A. Hydrogen bonds NH···N in compressed benzimidazole polymorphs. *Cryst. Growth Des.* **2013**, *13*, 696–700, DOI: 10.1021/cg301374z

(10) Obodovskaya, A. E.; Starikova, Z. A.; Belous, S. N.; Pokrovskaya, I. E. Crystal and molecular structure of 2-methylbenzimidazole. *Zh.Strukt.Khim.(Russ.)(J.Struct.Chem.)* **1991**, *32*, 421–422, DOI: 10.1007/BF00745764

(11) Zieliński, W.; Katrusiak, A. Colossal monotonic response to hydrostatic pressure in molecular crystal induced by a chemical modification *Cryst. Growth Des.* **2014**, *14*, 4247–4253, DOI: 10.1021/cg5008457

(12) Lee, Y.; Scheidt. W. R. Structure of 5,6-dimethylbenzimidazole. *Acta Cryst.* **1986**, *42*, 1652–1654, DOI: 10.1107/S0108270186091102

(13) Bondi, A. van der Waals Volumes and Radii. *J. Phys. Chem.* **1964**, *68*, 441– 451, DOI: 10.1021/j100785a001
